# An Effective, Green Synthesis Procedure for Obtaining Coumarin–Hydroxybenzohydrazide Derivatives and Assessment of Their Antioxidant Activity and Redox Status

**DOI:** 10.3390/antiox12122070

**Published:** 2023-12-01

**Authors:** Edina H. Avdović, Žiko Milanović, Dušica Simijonović, Marko Antonijević, Milena Milutinović, Danijela Nikodijević, Nenad Filipović, Zoran Marković, Radiša Vojinović

**Affiliations:** 1 Department of Science, Institute for Information Technologies, University of Kragujevac, Jovana Cvijića bb, 34000 Kragujevac, Serbia; ziko.milanovic@uni.kg.ac.rs (Ž.M.); dusicachem@kg.ac.rs (D.S.); mantonijevic@uni.kg.ac.rs (M.A.); zmarkovic@uni.kg.ac.rs (Z.M.); 2 Department of Biology and Ecology, Faculty of Science, University of Kragujevac, Radoja Domanovića 12, 34000 Kragujevac, Serbia; milena.milutinovic@pmf.kg.ac.rs (M.M.); danijela.nikodijevic@pmf.kg.ac.rs (D.N.); 3 Faculty of Engineering, University of Kragujevac, Sestre Janjic 6, 34000 Kragujevac, Serbia; 4 Department of Natural Science and Mathematics, State University of Novi Pazar, 36300 Novi Pazar, Serbia; 5 Faculty of Medical Sciences, University of Kragujevac, Svetozara Markovića 69, 34000 Kragujevac, Serbia; rhvojinovic@gmail.com

**Keywords:** coumarins, green synthesis, antioxidant activity, redox status, cancer therapy

## Abstract

In this study, green synthesis of two derivatives of coumarin–hydroxybenzohydrazide, (*E*)-2,4-dioxo-3-(1-(2-(2,3,4-trihydroxybenzoyl)hydrazyl)ethylidene)-chroman-7-yl acetate (**C–HB_1_**), and (*E*)-2,4-dioxo-3-(1-(2-(3,4,5-trihydroxybenzoyl)hydrazyl)ethylidene)chroman-7-yl acetate (**C–HB_2_**) is reported. Using vinegar and ethanol as a catalyst and solvent, the reactions were carried out between 3-acetyl-4-hydroxy-coumarin acetate and corresponding trihydroxybenzoyl hydrazide. The antioxidant potential of these compounds was investigated using the DPPH and ABTS assays, as well as the FRAP test. The obtained results reveal that even at very low concentrations, these compounds show excellent radical scavenging potential. The IC_50_ values for C-HB_1_ and C-HB_2_ in relation to the DPPH radical are 6.4 and 2.5 μM, respectively, while they are 4.5 and 2.0 μM in relation to the ABTS radical. These compounds have antioxidant activity that is comparable to well-known antioxidants such as gallic acid, NDGA, and trolox. These results are in good correlation with theoretical parameters describing these reactions. Moreover, it was found that inhibition of DPPH^●^ follows HAT, while inactivation of ABTS^+●^ follows SET-PT and HAT mechanisms. Additionally, coumarin–hydroxybenzohydrazide derivatives induced moderate cytotoxic activity and show significant potential to modulate redox status in HCT-116 colorectal cancer cells. The cytotoxicity was achieved via their prooxidative activity and ability to induce oxidative stress in cancer cells by increasing O_2_˙^−^ concentrations, indicated by increased MDA and GSH levels. Thus, ROS manipulation can be a potential target for cancer therapies by coumarins, as cancer cells possess an altered redox balance in comparison to normal cells. According to the ADMET analysis, the compounds investigated show good pharmacokinetic and toxicological profiles similar to vitamin C and gallic acid, which makes them good candidates for application in various fields of industry and medicine.

## 1. Introduction

The oxygen molecule plays a crucial role as an electron acceptor in cellular metabolism. It is a key component in cellular respiration, specifically oxidative phosphorylation. This molecule plays a vital role in the creation of energy as a part of the electron transport chain, which is involved in nearly all electron transfer processes in living organisms. Despite its many benefits, it can also facilitate the development of partially reduced chemical species called reactive oxygen species (ROS) [1,2]. ROS are capable of initiating a chain reaction that could be harmful to the cell. The body’s normal generation of ROS facilitates the immune system’s removal of foreign bodies from the blood, regulates cell signaling, hastens the ageing process, and so on. Drugs, air pollutants, UV rays, ionizing radiation, smoking, and some endogenous metabolites of the redox and respiratory chain all contribute to the continual production of ROS within the cells during the transfer of electrons.

Under normal circumstances, the organism can regulate the generation of ROS in such a way that the level of cellular damage caused by reactive species’ activity is kept to a minimum. This allows the organism to function normally. To protect itself from the damaging effects of oxidative stress, the human body has evolved a multifaceted defense mechanism that comprises preventative, antioxidant, and physiological defense [3]. Enzymatic and nonenzymatic antioxidants are both components of the body’s natural defense system against oxidative damage. The limited number of proteins that make up enzymatic antioxidants, such as catalase, glutathione peroxidase, and superoxide dismutase, are joined by a small number of enzymes that serve as auxiliary components. In the presence of cofactors such as copper, zinc, manganese, and iron, they convert reactive species into hydrogen peroxide, which is then converted into water. In accordance with the mode of operation, antioxidants can be split into two distinct subgroups: direct and indirect. When it comes to protecting the body from the damaging effects of oxidative stress, direct-acting antioxidants play a critical role. The vast majority of them enter the body from the food we eat, and only a small percentage are actually manufactured by the body itself. Vitamins, polyphenols, carotenoids, and other antioxidants are included in this category. The vast majority of antioxidants that occur in nature can be discovered in plants traditionally employed in medicine. Extraction, identification, and evaluation of the biological activity of these compounds have been very popular in the last decades [4,5,6,7]. It should be noted that scientists have spent years working on the development of new and effective antioxidant compounds that meet additional criteria such as better solubility, especially in water, and selective biological toxicity.

Natural coumarins are low-molecular-weight phenolic compounds containing a carbonyl group attached to C-2 of the γ-pyrone ring. They have been found in different essential oils, such as lavender oil, cinnamon bark oil, and cassia leaf oil. They are most prevalent in the fruits of higher plants, followed by the roots, stems, and foliage [8,9,10,11,12,13]. It should be noted that cinnamon contains more coumarins than any other victuals [14]. Important biological roles for coumarins include regulating growth, controlling respiration, and providing defense against herbivores and microbes. Coumarins act as hormones and signal molecules in a variety of biological processes. They have been studied extensively over the past decade for their potential antioxidant, anti-inflammatory, anticoagulant, enzyme-inhibitory, antibacterial, and anticancer effects. It should be noted that some of them found clinical use a long time ago, such as the antibiotics novobiocin and armilarizin A, anticoagulants warfarin, phenprocoumon, and acenocoumarol, and antispasmodic and choleretic hymecromone [8,9,10,11,12,13,14,15,16,17].

Condensation of 3-acetyl-4-hydroxycoumarin with suitable hydrazides under reflux in n-propanol or heating in ethanol with acetic acid as a catalyst yielded coumarin-hydrazide derivatives. A few studies [18,19,20] describe reactions involving 3-acetyl-4-hydroxycoumarin and other benzoyl hydrazides or primary amines. The formation of a new N-C bond occurs by nucleophilic attack, with the electron pair of the primary amino group participating exclusively in the formation of this bond. However, the use of benzoyl hydrazide with a pyrogallol fragment in the synthesis of these derivatives cannot be found in the literature. It should be emphasized that this study shows for the first time the use of 2,3,4-trihydroxybenzohydrazide and 3,4,5-trihydroxybenzohydrazide to obtain the corresponding coumarin derivatives.

The synthesis of novel coumarin hydroxybenzohydrazide derivatives that are anticipated to exhibit high levels of antioxidant and anticancer activity is the objective of this research. The method that was used for this synthesis was environmentally friendly, simple, and inexpensive. After the compounds had been synthesized, structural characterization and an investigation into their antiradical activity in vitro, their anticancer potential on human colorectal HCT-116 cancer cells, and their impact on redox status in cancer cells (superoxide anion radical, lipid peroxidation and glutathione level) was carried out.

## 2. Materials and Methods

### 2.1. Chemical Reagents and Instruments

Chemicals (purity > 98%) used for synthesis, characterization, assessment of antioxidant activity, and assessment of redox status of coumarin–hydroxybenzohydrazide derivatives: 4,7-dihydroxycoumarin, 2,3,4-trihydroxybenzoic acid, 3,4,5-trihydroxybenzoic acid, hydrazine monohydrate, phosphorus oxychloride, dimethyl sulfoxide (DMSO-*d*_6_), 2,2-diphenyl-1-picrylhydrazyl (DPPH), 2,2′-azino-*bis* (3-ethylbenzothiazoline-6-sulfonic acid (ABTS)), potassium persulfate, iron(III) chloride, potassium ferricyanide, trichloroacetic acid, nordihydroguaiaretic acid (NDGA), gallic acid, ascorbic acid, 5-(diethoxyphosphoryl)-5-methyl-1-pyrroline-*N*-oxide (DEPMPO), ethidium bromide, nitro blue tetrazolium chloride, 5,5′-dithio-*bis*(2-nitrobenzoic acid), trichloroacetic acid, thiobarbituric acid were purchased from Sigma-Aldrich Chemical Co. An immortalized adherent colorectal carcinoma cell line (HCT-116) was obtained from the American Type Culture Collection, ATCC. The chemicals methanol, ethanol, acetonitrile, toluene, acetone, acetic and hydrochloric acid, were purchased from a domestic producer.

The infrared spectra of coumarin–hydroxybenzohydrazide compounds were recorded with a PerkinElmer FT-IR spectrophotometer using the standard KBr technique in the range 4000–400 cm^−1^. NMR spectra (^1^H (200 MHz) and ^13^C NMR (50 MHz)) of these synthesized compounds were recorded using a Varian Gemini 200 NMR spectrophotometer, using deuterated DMSO-*d*_6_ as solvents. The UV-vis measurements were recorded on PerkinElmer, Lambda 365 UV/Vis spectrophotometer. The chemical elements carbon, hydrogen, and nitrogen were quantified in the samples using a CHNS/O elemental analysis system NC Technologies, ECS 8020 CHNOS.

### 2.2. Synthesis of Coumarin–Hydroxybenzohydrazide Derivatives

**Scheme 1:**
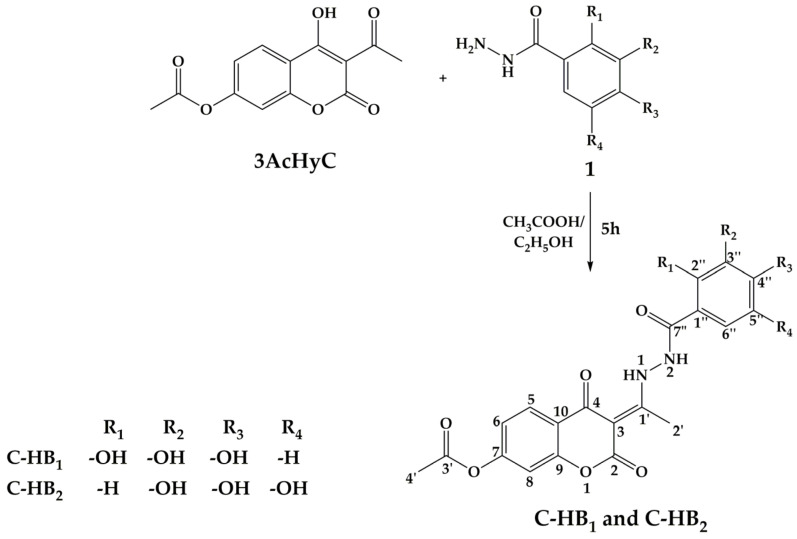
Synthesis of coumarin–hydroxybenzohydrazide derivatives (**C–HBs**).

Compounds: (E)-2,4-dioxo-3-(1-(2-(2,3,4-trihydroxybenzoyl)hydrazyl)ethylidene) chroman-7-yl acetate (**C–HB_1_**) and (E)-2,4-dioxo-3-(1-(2(3,4,5-trihydroxybenzoyl)hydrazyl)ethylidene)-chroman-7-yl acetate (**C–HB_2_**), were obtained in the reaction of previously synthesized 3-acetyl-4-hydroxy-2-oxo-2H-chromen-7-yl acetate (**3AcHyC**) and hydroxybenzohydrazines (2,3,4-trihydroxybenzohydrazide and 3,4,5-trihydroxybenzohydrazide) [20]. The hydroxybenzohydrazines **1** (0.001 mol) and 3AcHyC (0.001 mol) were dissolved in a mixture of vinegar and ethanol (1:1) (20 mL) and stirred at reflux for 5 h (80 °C). The course of the reaction was followed by thin-layer chromatography, where a mixture of ethyl acetate and dichloromethane in a ratio of 2:1 was used as the eluent (Scheme 1). When the reaction was completed, the obtained mixture was cooled to room temperature and the precipitate was collected by filtration, and then the obtained products were recrystallized from ethanol (Scheme 1)

(*E*)-2,4-dioxo-3-(1-(2-(2,3,4-trihydroxybenzoyl)hydrazyl)ethylidene)chroman-7-yl acetate (**C–HB_1_**). Yield: 0.309 g (72.2%). Calculated (C_20_H_16_N_2_O_9_, Mr = 428.09): C, 56.08%; H, 3.77%; N, 6.54%, O, 33.66%. Found: C, 56.15%; H, 4.00%; N, 6.32%, O, 33.53%. ^1^H NMR (DMSO-*d*_6_, 200 MHz), δ ppm: 2.31 (3H, s, C4′–H), 2.65 (3H, s, C2′–H), 6.57 (1H, m, C5″–H), 6.83 (1H, s, C8–H), 7.27 (1H, s, C6–H), 7.84 (1H, m, C6″-H), 8, 73 (1H, d, 3JH–5,H–6 = 8.5 Hz, C5–H), 9.93 (1H, s, O4″–H), 10.67 (1H, s, O3″–H), 11.57 (1H, s, O2″–H), 11.29 (1H, s, N2–H), 15.57 (1H, s, N1–H). ^13^C NMR (DMSO-*d*_6_, 50 MHz), δ ppm: 17.4 (C2′), 29.4 (C4′), 98.7 (C3), 102.1 (C1″), 106.5 (C5″), 107.7 (C8), 111.8 (C6″), 112.8 (C10), 114.1 (C6), 119.5 (C5), 127.3 (C3″), 127.5 (C4″), 133.0 (C2″), 149.6 (C9), 150.8 (C7), 155.0 (C1), 163.1 (C7″), 166.6 (C3′), 171.4 (C4), 178.9 (C1′). IR (KBr), cm^−1^: 3691, 3193 (O–H/C–H/N–H), 1658, 1614 (C=O), 1555, 1444 (C–C), 1328, 1283, 1165 (C–O) cm^−1^.

(*E*)-2,4-dioxo-3-(1-(2-(3,4,5-trihydroxybenzoyl)hydrazyl)ethylidene)chroman-7-yl acetate (**C–HB_2_**). Yield: 0.326 g (76.2%). Calculated (C_20_H_16_N_2_O_9_, Mr = 428.09): C, 56.08%; H, 3.77%; N, 6.54%; O, 33.66%. Found: C, 56.10%; H, 4.10%; N, 6.50%, O, 33.30%. ^1^H NMR (DMSO-*d*_6_, 200 MHz), δ ppm: 2.31 (3H, s, C4′–H), 2.85 (3H, s, C2′–H), 6.95 (1H, m, C2″–H, C6″–H), 7.13 (2H, m, C6–H, C8–H), 8.01 (1H, d, 3JH–5,H–6 = 8.5 Hz, C5–H), 9.87 (3H, s, O3″–H, O4″–H, O5″–H), 11.48 (1H, s, N2–H), 15.67 (1H, s, N1–H). ^13^C NMR (DMSO-*d*_6_, 50 MHz), δ ppm: 17.9 (C2′), 21.1 (C4′), 94.9 (C3), 107.1 (C2″, C6″), 109, 9 (C8), 117.7 (C10), 118.1 (C6), 120.8 (C1″), 127.1 (C5), 138.2 (C4″), 145.9 (C3”), 149.5 (C5″), 153.8 (C9), 154.8 (C7), 161.5 (C1), 165.1 (C7″), 168.7 (C3′), 171.4 (C4), 178.7 (C1′). IR (KBr): 3387, 3157, 2981, 2938 (O–H/C–H/N–H), 1732, 1698, 1642 (C=O), 1556, 1506, 1465, 1365 (C–C), 1329, 1258, 1145 (C–O) cm^−1^.

### 2.3. In Vitro Tests for the Assessment of Antioxidant Activity

#### 2.3.1. DPPH Radical Scavenging Assay

The ability of coumarin–hydroxybenzohydrazide compounds to inactivate reactive radical species was determined using the DPPH test [21]. In more detail, the investigated compounds (20 μL of different concentrations (25, 50, 100 μM) dissolved in DMSO and 980 μL methanol) were mixed with an equal volume of DPPH solution in methanol (1000 μL; 0.05 mM). The prepared samples were well shaken and left at room temperature in the dark for 30 and 60 min. After incubation, absorbance at 517 nm was measured using methanol as a blank probe. All measurements were performed in triplicate, and the results were presented as mean ± SD (standard deviation) [22]. After that, the IC_50_ values were also determined, i.e., compound concentrations necessary to generate 50% reduced DPPH^•^. The stoichiometric factor (SF) was calculated using the following equation [22]:

(1) $$SF=\frac{{\left[DPPH\right]}_{0}}{\left(2\times IC_{50}\right)}$$

NDGA and gallic acid were used as positive controls.

#### 2.3.2. ABTS Radical Scavenging Assay

The first step in calculating the percentage of reduction in this test is to prepare a basic 7 mM ABTS solution mixed with 2.45 mM potassium persulfate (K_2_S_2_O_8_) solution 16 h before the experiment to form the radical cation ABTS^•+^. The ABTS^•+^ stock solution was then diluted with methanol until the absorbance at wavelength = 734 nm was 0.70. Different concentrations of samples were prepared in DMSO. Following that, 20 μL of the sample and 980 μL of methanol were mixed, and an equal amount of previously produced ABTS solution was added before measuring the absorbance at 734 nm. The antioxidant capacity is expressed through the IC_50_ value, i.e., compound concentrations necessary to generate 50% reduced ABTS^•+^ [23].

#### 2.3.3. Ferric Reducing Antioxidant Power (FRAP) Assay

The sample is prepared by dissolving the test compound in an appropriate organic solvent (DMSO), and its initial concentration should be approximately 1.2 mM and the absorbance value at a wavelength of 700 nm should not be greater than 1. Phosphate buffer (PBS) pH = 7.4 and 1% K_3_[Fe(CN)_6_] was used for sample preparation. Measurements were performed in triplicate, namely, 20 μL of the tested compound of initial concentration, 480 μL of PBS and 250 μL of 1% K_3_[Fe(CN)_6_] and incubated for 20 min at 50 degrees. Then, weigh out 500 μL of the incubated sample and add 500 μL of 10% Cl_3_CCOOH, 100 μL of 0.1% FeCl_3_ and 500 μL of H_2_O. After 10 min of incubation at room temperature, measure the absorbance at 700 nm [24].

### 2.4. In Vitro Determination of Anticancer Activity

#### 2.4.1. Cytotoxic Effects of Coumarins

The cytotoxicity of coumarins on human colorectal carcinoma cell viability (HCT-116) was examined by MTT assay [25]. After 24 h of seeding in a 96-well plate (10^4^ cells/well), HCT-116 cells were treated with 100 µL of coumarins (**C–HB_1_**, **C–HB_2_**) in a concentration range of 1–250 µM. In control cells, the medium was replaced, and microplates were incubated for 24 and 72 h. The reaction between mitochondrial dehydrogenase and yellow MTT produces purple colored formazan that dissolves in 150 µL of DMSO per well. Absorbances were obtained at 550 nm (ELISA reader). Results are shown as the percentage of viable cells in all applied concentrations. IC_50_ values were obtained from the viability curves (by the CalcuSyn program).

#### 2.4.2. Effects of Coumarins on Redox Status in Cancer Cells

##### Superoxide Anion Level (O_2_˙^−^)

The concentration of superoxide anion radical (O_2_˙^−^) in HCT-116 control and coumarin-treated cells was determined by NBT assay [26]. After 24 h of seeding in a 96-well plate (10^4^ cells/well), HCT-116 cells were treated with 100 µL of **C–HB_1_** and **C–HB_2_** in a concentration range of 1–250 µM. After 24 h of treatment incubation, 10 μL of NBT solution (in concentration of 5 mg/mL), was added to the each well and incubated for 45 min at 37 °C. After incubation, 10 μL of DMSO was added to each well. The absorbances were obtained at 550 nm (ELISA reader). The concentration of O_2_˙^−^ was expressed in nmol/mL, according to the formula: nmol NBT/mL = A/0.015 × Vcuv/Vex (A—absorbance; Vcuv—total volume of solution in the well = 120 μL; Vex—cell volume with treatment = 100 μL; 0.015—molar extinction coefficient for monoformazan (15,000 M^−1^ cm^−1^). The results were then calculated according to the number of viable cells previously obtained in all applied concentrations by MTT.

##### Damage to Membrane Lipids

The concentration of malondialdehyde (MDA), an indicator of membrane lipid peroxidation under the influence of coumarins, was determined by TBARS assay [27]. After 24 h of cell seeding in a 6-well plate (106 cells/well), HCT-116 cells were treated with 100 µL of **C-HB_1_** and **C-HB_2_** in a concentration range of 1–250 µM. After 24 h of treatment incubation, the cells were centrifuged and the supernatant was used for the following steps. For each sample, 10 µL was separated for protein concentration measurements on the biophotometer (protein concentration was determined based on the standard curve factor). The rest of the supernatant was mixed with 1 mL of the reaction mixture TCA-TBA-HCl, which contains 15% trichloroacetic acid, 0.375% thiobarbituric and 0.25 M hydrochloric acid. Samples were heated to 90 °C, for 30 min, refrigerated for 5 min on ice, and then centrifuged for 10 min at 6600 rpm, 4 °C. After that, the supernatant was transferred to 96-well plates (100 µL per well) and absorbance was measured at 405 nm (ELISA reader). The concentration of MDA (pmol/mg of protein) was calculated by the previously described formula [27]. The results were then calculated according to the number of viable cells previously obtained in all applied concentrations by MTT.

##### Glutathione Level (GSH)

The concentration of GSH under the influence of coumarins was determined by the colorimetric method [28]. After 24 h of cell seeding in a 96-well plate (5 × 10^4^ cells/well), HCT-116 cells were treated with 100 µL of **C–HB1** and **C–HB2** in a concentration range of 1–250 µM. After 24 h of treatment incubation, the plate was centrifuged at 1000 rpm for 10 min. After centrifugation, in each well, the medium was replaced with 100 μL 2.5% sulfosalicylic acid and incubated on ice for 15 min. After that time, the plate was centrifuged for 15 min at 1000 rpm, and then the 50 μL of each sample was added to the new microtiter plate in triplicate. To each sample was added 100 μL of the reaction mixture, which contains 1 mM NADPH, 0.7 U GSH reductase per milliliter of the reaction mixture, 1 mM DTNB, dissolved in DMSO and supplemented with PBS to the required volume. The absorbances were obtained at 405 nm (ELISA reader). The concentration of GSH was expressed in nmol/mL (related to the standard curve constituted of known molar BSA concentrations). The results were then calculated according to the number of viable cells previously obtained in all applied concentrations by MTT.

### 2.5. Computational Methodology

All calculations were performed using the Gaussian09 software program [29]. The optimization of the structures of parent molecules, as well as their corresponding radicals, anions, and radical cations, was performed using quantum chemical calculations. Specifically, the M06-2X functional in combination with the 6-311++G(d,p) basis set, which includes polarization and diffuse functions, was employed [30,31]. The CPCM continuous solvation model [32] was employed to computationally simulate the molecular structures in methanol (ε = 32.61) without any geometrical constraints. No imaginary frequencies were found, indicating that the structures obtained were found at energy minima on the potential energy surface. The selection of this solvation model was based on its ability to accurately simulate polar conditions and the environment in which the experimental measurements were carried out. The chemical shifts of the examined compounds in NMR spectra were estimated using the gauge independent atomic orbital (GIAO) method. The ADMETlab 2.0 web server was employed to assess the toxicity profile and significant pharmacokinetic features of ADMET (absorption, distribution, metabolism, excretion, toxicity) analysis [33].

### 2.6. Statistical Analysis

All data are expressed as mean ± standard error (SE) and were gained from two individual experiments. Statistical significance was determined by the one-way ANOVA test for multiple comparisons. A *p*-value < 0.05 was considered significant. The magnitude of the correlation between variables was calculated using software (SPSS for Windows, ver. 17, 2008, Chicago, IL, USA).

## 3. Results and Discussion

### 3.1. Synthesis and Structural Characterization of Novel Coumarin–Hydroxybenzohydrazide Derivatives (C-HB_1_ and C-HB_2_)

Bearing in mind the fact that antioxidants have a very important role in the prevention of diseases caused by free radical damage, the goal of this study was to synthesize and structurally characterize coumarin–hydroxybenzohydrazide compounds with pyrogallol moiety. These compounds were obtained in the reaction between 3-acetyl -4-hydroxy-2-oxo-2*H*-chromen-7-yl acetate and corresponding trihydroxybenzohydrazide by using a mixture of vinegar and ethanol as a catalyst and solvent. After 5 h of reflux, the pure reaction products were separated in good yield (72 and 76%) without purification.

Coumarin–hydroxybenzohydrazide compounds **C–HB_1_** and **C–HB_2_** were characterized based on NMR (^1^H and ^13^C), IR spectral properties, and elemental analysis. The experimental and simulated chemical shifts in the ^1^H NMR and ^13^C NMR spectra (Figures S1 and S2), measured relative to TMS as an internal standard, can be found in Tables S1 and S2. The obtained factors of correlation (R) for ^1^H NMR and ^13^C NMR, which are greater than 0.993, along with the low values of mean absolute error, MAE (<1.4 for ^1^H NMR and <8.13 for ^13^C NMR), suggest that the theoretical model employed effectively described the structural characteristics of the newly synthesized compounds.

In the ^1^H NMR spectra of **C–HB_1_** and **C–HB_2_**, three distinct proton groups can be distinguished. The first group consists of protons originating from methyl groups C2′ and C4′, which appear in the spectrum as distinct singlets. The chemical shifts of the observed signals range from 2.31 to 2.85 ppm in the experimental spectra, while in the theoretical spectra, they range from 2.32 to 2.79 ppm (Table S1). Due to the influence of the adjacent *sp^2^* hybridized carbon atom and the proximity of numerous carbonyl and secondary amino groups, the proton of the C2′ methyl group has a slightly greater chemical shift.

The second group of protons belong to the aromatic ring of the coumarin base and the phenolic ring of the substituent. These protons are observed to resonate between 6.57 and 8.73 ppm in the experimental spectrum, and between 6.76 and 8.01 ppm in the theoretical spectrum (Table S1). The third group of protons comes from the polar hydroxyl and amino groups. In compound **C–HB_1_**, three signals originating from asymmetric –OH groups are distinguished, as well as two signals of protons originating from amino groups. Signals originating from protons O2″–H (11.57 ppm) and N1–H (15.57 ppm) are at the highest chemical shift due to stabilization by strong hydrogen bonds with neighboring oxygen atoms. In the ^1^H NMR spectrum of **C–HB_2_**, the symmetric protons of the polar –OH groups are found in the form of a singlet at 9.87 ppm, while the protons of the –NH group as in the case of **C–HB_1_** are located at higher chemical shifts. Our earlier results and the fact that the ^1^H NMR spectra of all synthesized compounds had a chemical shift at around 15 ppm, which may be attributable to the presence of the proton-NH group [34,35,36], support the validation of the structure of investigated compounds. Due to the interaction between protons of polar groups (–OH) and solvent molecules in a real system, the values obtained from the implicit solvation model simulations will be underestimated and lack practical significance.

In the analyses of ^13^C NMR spectra, the chemical shifts of carbon atoms can be divided into two groups. The first involves the sp^3^ hybridized carbon atoms from C2′ and C4′ methyl groups, with chemical shifts ranging from 17.4 and 29.4 ppm in experimental and between 13.9 and 17.3 in theoretical spectra (Table S2). The carbon atoms of the coumarin core and the phenolic ring make up the second group. The chemical shift values for the second group of carbon atoms cover a wide range between 94.9 and 178.9 ppm. As anticipated, the carbon atoms of the carbonyl groups C4, C3′, and C7″ have high chemical shift values. These values are explained by the *sp^2^* hybridization of the carbon atoms and the negative inductive effect of the oxygen atoms. Chemical shift values for C1′ are roughly 179 ppm, which is considerably higher than anticipated. Extensive delocalization between the coumarin core and the aromatic ring at position 3, as well as the presence of numerous electronegative nitrogen and oxygen atoms, contribute to the very high values observed.

**C-HB_1_** and **C-HB_2_** experimental IR spectra (Figures S3 and S4) were recorded in the region between 4000 and 400 cm^−1^. Due to the structural similarity of these compounds, no significant differences in wavenumber positions are expected, except for those modes affected by the position of aromatic phenolic –OH groups.

Three clearly defined regions can be observed in the obtained IR spectra. The characteristic bands of these spectra contribute to elucidating the structure of the investigated compounds.

The first region, between 4000 and 2800 cm^−1^, consists mostly of stretching vibrations. Broad bands can be observed at higher wavenumbers, which indicate the superposition of stretching vibrations from the O–H, C–H, and N–H bands. The wavenumbers for the broadband mentioned are 3193 (**C–HB_1_**) and 3387 (**C–HB_2_**) cm^−1^. In the second region, between 1800 and 1000 cm^−1^, a large number of peaks appear that can be attributed to a mixture of stretching and bending vibrations. This region starts with several intense bands belonging to C=O stretching vibrations. The wave numbers for C=O stretching vibrations are similar for both molecules and range from 1559 to 1776 cm^−1^. The ester carbonyl group (C3′=O) has the highest wavenumber, with values of 1658 (**C–HB_1_**) and 1698 (**C–HB_2_**) cm^−1^. The C2=O, C4=O, and C7″=O vibrations, on the other hand, are superimposed into intense bands with lower wavenumber values. Other intense bands with wavenumber values between 1300 and 1000 cm^−1^ include C–C and C–O stretching as well as C–C–H bending vibrations. The characteristic C-O vibration peaks are positioned at 1328, 1283, and 1165 cm^−1^ (**C–HB_1_**) and 1329, 1258, and 1145 cm^−1^ (**C–HB_2_**). Other intense bands with wavenumber values between 1300 and 1000 cm^−1^ include C–C and C–O stretching as well as C–C–H bending vibrations. The peaks of characteristic C–O vibrations are positioned between 1444 and 1555 cm^−1^ (**C–HB_1_**) and 1365 and 1556 cm^−1^ (**C–HB_2_**). The third region includes values < 1000 cm^−1^ and is characterized by peaks of medium to low intensity, attributed to bending and torsional vibrations or their combination with stretching vibrations.

### 3.2. In Vitro Radical Scavenging Ability of Coumarin–Hydroxybenzohydrazide Derivatives

It is known that some coumarins have good antioxidant potential. To determine the antioxidant capacity of coumarin derivatives **C–HB_1_** and **C–HB_2_**, combinations of different assays such as DPPH, ABTS and FRAP tests were used. As standards, the known antioxidants gallic acid, NDGA, trolox, and vitamin C were used. The obtained values of DPPH and ABTS tests are presented as IC_50_ values, which are defined as the effective concentration of antioxidants required to reduce by 50% the initial concentration of these radicals.

#### 3.2.1. Antioxidant Activity of C-HBs against DPPH Radical

The DPPH radical scavenging test is one of the most often used assays and is the primary step for assessing antioxidant activity. Thus, antioxidant molecules can neutralize DPPH free radicals by giving them hydrogen atoms or by donating electrons, which causes a decrease in absorbance at 517 nm [37].

Preliminary evaluation for DPPH inhibitory activity of coumarin–hydroxybenzohydrazide compounds, as well as the standards of gallic acid, NDGA (according to the DPPH), was conducted at 25 µM, 50 µM, and 100 µM, and for incubation times of 20 and 60 min, as shown in Table 1. The activity of these bioactive compounds is significant, even at low concentrations of 25 µM, reaching 92% for **C–HB_1_** and **C–HB_2_**. As a consequence, even lower concentrations were utilized in the calculations of IC50 values; the resulting values are presented in Table S3. Based on certain IC_50_ values, more precise information about antioxidant capacity can be obtained. The IC_50_ values against the DPPH radical for **C–HB_1_** and **C–HB_2_** are 6.4 and 2.5 µM. The activity of **C–HB_2_** compound is comparable to the activity of standard good antioxidants such as gallic acid (IC_50_ = 2.6 µM) and NDGA (IC_50_ = 1.7 µM). The stoichiometric factor (SF) value of this compound, which is 5.0, indicates very good antioxidant activity, taking into account the fact that very good scavengers of free radicals have an SF > 2 [19]. The second compound **C–HB_1_** is a slightly weaker antioxidant because its IC_50_ is 6.4 µM, while its SF is 2 (Table 1).

By comparing the antioxidant potential of these compounds with similar 4-hydroxycoumarin derivatives, which contain one or two hydroxyl groups, or some other substituent in their structure, a significant difference was revealed. Namely, the antioxidant capacity of the investigated compounds with three OH groups (**C–HB_1_** and **C–HB_2_**) is significantly stronger. This fact is explained by the presence of additional OH groups, which significantly promote the formation of stable phenoxy radical, which is believed to play a critical role in radical scavenging activity [19,20]. The structure–activity relationship clearly shows that phenolic groups are essential for antioxidant activity.

#### 3.2.2. Antioxidant Activity of C-HBs against ABTS Radical Cation

The antioxidant capacity of the tested compounds **C–HB_1_** and **C–HB_2_** and trolox as a reference antioxidant was determined according to the ABTS^•+^. It has already been emphasized that the test is based on the reduction of previously generated ABTS^•+^ to a neutral form whose percentage reduction is determined based on the decrease in absorbance in the absorption spectrum. The results are expressed in terms of percentage inhibition and IC_50_ values (Table 2).

The data in the Table 2 show that the compounds’ activity towards the ABTS^•+^ radical is similar to what was seen with the DPPH radical. Both compounds demonstrated an exceptional capacity to neutralize ABTS^•+^, with IC_50_ values approximately 1.2 to 2.7 times lower than trolox (5.3 µM). **C-HB_2_** exhibits activity of 20.4–68.9% at concentrations between 1.0 and 3.0 µM, whereas **C–HB_1_** at higher concentrations between 4.0 and 8.0 µM displays activity between 47.9% and 64.7%. In this instance as well, **C–HB_2_** exhibits superior antioxidant activity, as indicated by its lower IC_50_ value.

#### 3.2.3. Ferric Reducing Antioxidant Power of C-HBs

The FRAP method [38] is based on the reduction of the Fe^3+^–ferricyanide complex to the ferrous form (Fe^2+^). Compounds **C–HB_1_** and **C–HB_2_** were tested for their reducing power through the reaction with the complex potassium ferricyanide. The results of investigated compounds and the well-known antioxidant ascorbic acid are shown as absorbance at 700 nm (Table 3). Table 3 shows the absorbance values of substances that can turn Fe^3+^ ions into Fe^2+^ ions. The strong redox potential of the compound is indicated by an increase in absorbance. Therefore, based on the absorbance for **C–HB_1_** (0.3979) and **C–HB_2_** (0.7725), it can be said that these compounds have a strong reduction potential that is much higher than the reduction potential of ascorbic acid (0.1249).

### 3.3. Anticancer Properties of Coumarin–Hydroxybenzohydrazide Derivatives

#### 3.3.1. Cytotoxicity

**Figure 1:**
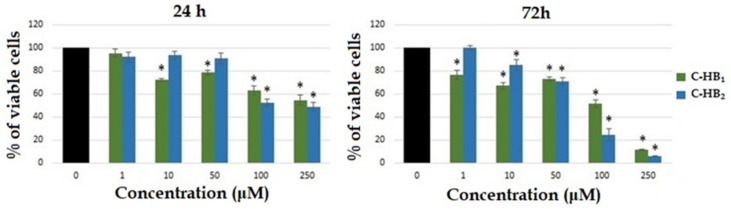
Effects of coumarins on HCT-116 cell viability, after 24 and 72 h. Results are presented as means ± SE of three independent experiments. * Statistically significant difference (*p* ˂ 0.05) in relation to control values. The black bar represents the control group.

Both investigated coumarins induce inhibition of HCT-116 cell proliferation 24 and 72 h after treatment (Figure 1). Inhibition of cell growth was significant in all applied concentrations in treatment with **C–HB_1_** for both investigated periods, as well as for **C–HB_2_** for 72 h, while the significant and at the same time the strongest inhibition of cell growth by **C–HB_2_** was observed only at the highest concentrations (100 and 250 μM) at 24 h.

IC_50_ values, calculated from the viability curves, indicate good-to-moderate cytotoxicity of tested coumarins, depending on the type of coumarin and time of treatment exposure (Table 4). According to IC_50_ values, **C–HB_2_** induced stronger cytotoxicity, especially after 72 h. Generally, the coumarin–hydroxybenzohydrazides **C–HB_1_** and **C–HB_2_** showed good-to-moderate cytotoxic activity on HCT-116 colorectal cancer cells. Our results correlate with the results of other authors, where the many synthesized coumarin derivatives induce similar cytotoxicity on breast MDA-MB-231 and MCF-7 cells, colon SW-480 and cervix HeLa adenocarcinoma cells [16,39,40,41].

#### 3.3.2. Effects of Coumarins on Redox Status in Cancer Cells

Targeted therapy, which involves disrupted internal levels of free radicals, is very important in the treatment of inflammatory types of cancer like colorectal carcinoma. This is often achieved by a combination of chemotherapeutics and supplements that have the additional possibility to modulate the redox status in cancer cells. It was confirmed by many authors that cytotoxicity correlates with the ability of substances to induce oxidative stress in cancer cells. Mainly elevated concentrations of reactive oxygen or nitrogen species initiate apoptosis, a favorable type of induced cell death [42].

**Figure 2:**
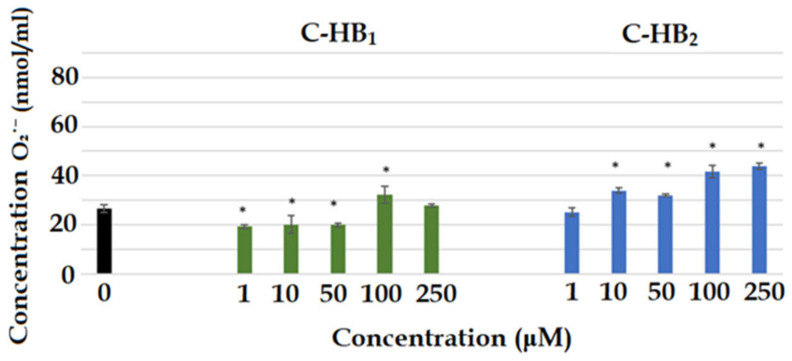
Effects of coumarins on O_2_˙^−^ concentration in HCT-116 cells after 24 h. Results are presented related to the number of viable cells, as means ± SE of three independent experiments. * Statistically significant difference (*p* ˂ 0.05) in relation to control values. The black bar represents the control group.

Results show that **C–HB_1_** induces prooxidative effects in HCT-116 cells via increasing of reactive oxygen species, O_2_˙^−^ concentration, in treated cell samples compared to control cells (Figure 2). These effects are also induced by **C–HB_2_** coumarin in the highest concentrations (100 and 250 μM), while the lower concentrations of **C–HB_2_** decreased the O_2_˙^−^ concentration and induce antioxidative properties.

**Figure 3:**
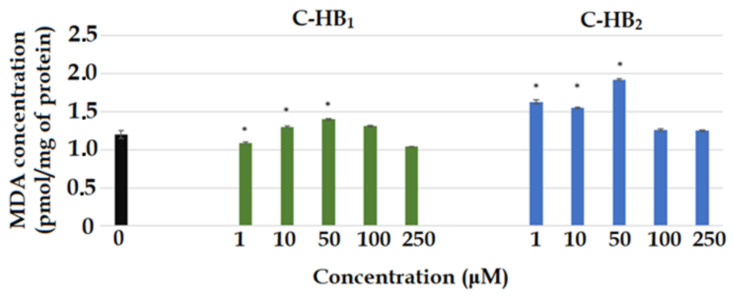
The effects of coumarins on level of lipid peroxidation, Malondialdehyde concentration expressed in pmol/mL of proteins in HCT-116 cells. Results are presented related to the number of viable cells, as means ± SE of three independent experiments. * Statistically significant difference (*p* ˂ 0.05) in relation to control values. The black bar represents the control group.

Malondialdehyde (MDA) concentration indicates lipid damage due to oxidative stress. It was detected/increased in **C–HB_1_** and **C–HB_2_** treated samples compared to control (Figure 3). The differences were observed in the effects of lower concentrations of two different coumarins, where the better effects achieve **C–HB_1_** compared to **C–HB_2_** at lower concentrations, as well as cytotoxicity.

**Figure 4:**
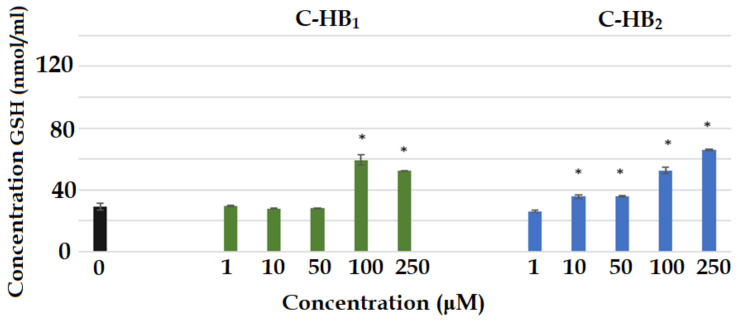
Effects of coumarins on GSH concentration (related to the number of viable cells) in HCT-116 cells after 24 h. Results are presented related to the number of viable cells, as means ± SE of three independent experiments. * Statistically significant difference (*p* ˂ 0.05) in relation to control values. The black bar represents the control group.

Following the determination of reactive oxygen species and presence of indicators of oxidative stress, the concentration of glutathione was measured as the first component of antioxidant protection in the fight against oxidative stress. Similar results were observed for glutathione concentration in treated HCT-116 cells. It was increased in treatment by **C–HB_1_** and in highest concentrations of **C–HB_2_** coumarin (Figure 4).

According to our results, coumarin–hydroxybenzohydrazide derivatives induced significant changes in redox status parameters regarding prooxidative effects in HCT-116 cancer cells via increasing O_2_˙^−^ concentrations, MDA as an indicator of lipid peroxidation and GSH level. These increases in biomarkers of oxidative stress correlate with achieved cytotoxic activity in appropriate concentrations. Also, there are correlations between increased O_2_˙^−^ concentrations and level of damaged lipids, as well as GSH levels that are elevated in high doses of treatment—as a response to oxidative stress. The lower concentrations of **C-HB_2_** were an exception to the trend of peroxidative effects, and they even acted as an antioxidant. This confirms the correlation between increased oxidative stress and cytotoxicity of coumarins, since those concentrations did not have a statistically significant effect on the inhibition of HCT-116 cell proliferation after 24 h. Khan et al. (2020) have reported similar results for synthesized derivate of coumarin (di(2-picolyl)amine-3(bromoacetyl)coumarin), which also increases the intracellular level of ROS, as well as damage of DNA and induction of apoptosis of hepatocellular carcinoma induced in rats. The increase in ROS has consequently led to an increase in the level of lipid peroxidation [43]. Other derivatives of coumarin, i.e., coumarin-di(2-picolyl)amine, have also induced ROS-dependent inhibition of cell growth, breast cancer MCF-7 and colon HCT-116 cells. As a chelate agent, it leads to an additional increase in copper in cancer cells, which directly affects the disruption of superoxide anion levels [44]. Due to such literature data, the examination of the activity of coumarin derivatives from the aspect of their influence on redox status parameters is fully justified.

### 3.4. Determination of the Plausible Mechanisms of Antiradical Activity of Coumarin–Hydroxybenzohydrazide Derivatives

**Figure 5:**
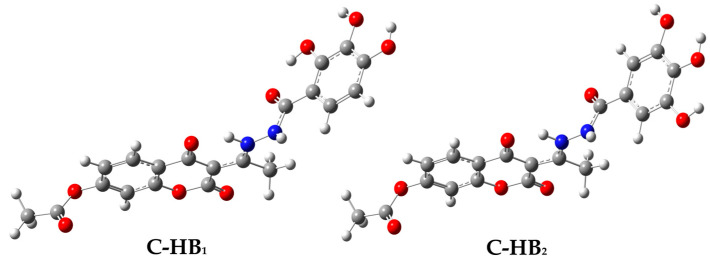
Equilibrium geometries of the investigated compounds obtained at M06-2X/6-311++G(d,p)/CPCM level of theory in methanol. Legend: gray—carbon atom, white—hydrogen atom, red—oxygen atom, blue—nitrogen atom. The atom numbering correlates with the numbering in Scheme 1.

The **C-HB_1_** and **C-HB_2_** compounds underwent investigation of the thermodynamic parameters of standard mechanisms of antioxidative activity, with the aim of accurately identifying the dominant mechanism. The study involved examining standard mechanisms of antiradical activity using reactive DPPH^•^ and ABTS^•+^ radical species. The investigations were carried out using methanol as the solvent to mimic the conditions of the experimental measurements. The optimized geometries of the newly synthesized compounds were calculated using the M06-2X/6-311++G(d,p)/CPCM theoretical model in methanol as solvent (Figure 5). The geometries of the compounds under investigation were elucidated by establishing a correlation with the geometries of structurally related compounds acquired in the previous research [19,20].

The analysis of the presented geometries reveals that the compounds under investigation participate in the deactivation of radical species via –OH groups and N2–H atoms. The N1–H group was excluded from further analysis due to the significant intramolecular stability provided by the hydrogen bond, as well as the resulting geometry distortion and limited electron delocalization in formed radical species obtained after hydrogen abstraction from the N1–H group.

#### 3.4.1. Investigation of Radical Scavenging Mechanism towards DPPH Radical

**Figure 6:**
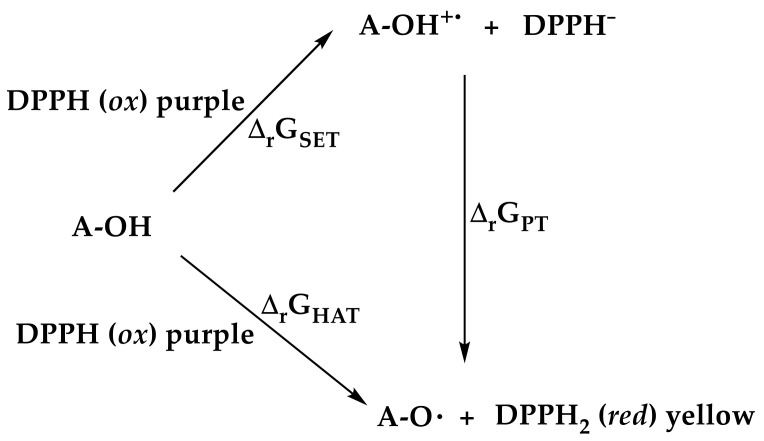
The investigation reaction mechanisms—hydrogen atom transfer (HAT) and Single electron transfer followed by proton transfer (SET-PT)—between 2,2-diphenyl-1-picrylhydrazyl (DPPH^•^) radical and **C-HB_1_**/**C-HB_2_** (A-OH).

Figure 6 illustrates the plausible reaction pathways that may occur between the investigated compounds **C-HB_1_**/**C-HB_2_** (A–OH) and DPPH radical: hydrogen atom transfer (HAT) and single electron transfer followed by proton transfer (SET-PT). Both mechanisms were shown to result in the generation of less reactive radical species (A–O^•^) for the compounds under investigation, as well as the production of the reduced DPPH radical.

The utilization of CPCM method, simulating methanol as a solvent, for all calculations mimicked the conditions of in vitro DPPH measurements. The reaction free energies (Δ_r_*G*), which quantify the energy difference between reactants and products, serve as the primary factor for assessing the probability of reaction mechanisms playing out. Upon examination of the data presented in Table 5, it is evident that both **C-HB_1_** (159 kJ mol^−1^) and **C-HB_2_** (165 kJ mol^−1^) had endergonic ∆_r_*G*_SET_ values. Despite the distinctly exergonic values for the second step of the mechanism, the first step demonstrates endergonic and unfavorable values, suggesting that the investigated compounds do not exert their antiradical activity through the SET-PT mechanism.

The reactivity of different positions of the **C–HB_1_** compound, when subjected to a direct reaction with the DPPH radical, follows a decreasing trend as follows: **3-OH > 2-OH > N2-H > 4-OH**. Conversely, the reactivity of various positions within the **C–HB_2_** compound, when undergoing a direct reaction with the DPPH radical, follows a decreasing trend as follows: **4-OH > 3-OH/5-OH > N2-H.** The discussion of geometries and spin density distribution in formed radical species (Figure S5) provides an elucidation of the reactivity of the position of the examined compounds.

The increased reactivity observed at the 3-OH position of compound **C–HB_1_** and the 4-OH position of compound **C–HB_2_** can be attributed to the stabilization of the resultant radical species by intramolecular hydrogen bonds. It can be concluded that these positions determine the reactivity of the investigated compounds. Therefore, based on the slightly exergonic ∆_r_*G*_HAT_ values, it can be observed that the 4-OH (−1 kJ mol^−1^) position of **C-HB_2_** compound has a slightly greater capacity to deactivate the DPPH radical compared to the 3-OH (9 kJ mol^−1^) position of the **C-HB_1_** compound (Table 5). This claim is supported by the experimentally validated reactivity observed through in vitro assessment of the DPPH activity of the investigated compounds. The formation of radicals at position 2-OH of the **C-HB_1_** compounds is thermodynamically favorable, primarily because radical species are stabilized by intramolecular hydrogen bonds N2-H······O2 and O3-H······O2. This stabilization occurs because of the free rotation of the C7″-C1″ bond following the removal of the hydrogen atom. Ultimately, by the process of abstracting the hydrogen atom from the N–H group in both compounds, radical species with planar geometry are generated. Furthermore, it can be observed that the spin density predominantly resides on the nitrogen atom (>0.462 *e*), rendering this position less favorable for interaction with the DPPH^•^. It makes sense to assume that the DPPH^•^ in methanol will be scavenged through the HAT mechanistic pathway considering the previously provided information.

#### 3.4.2. Investigation of Radical Scavenging Mechanism towards ABTS Radical Cation

**Figure 7:**
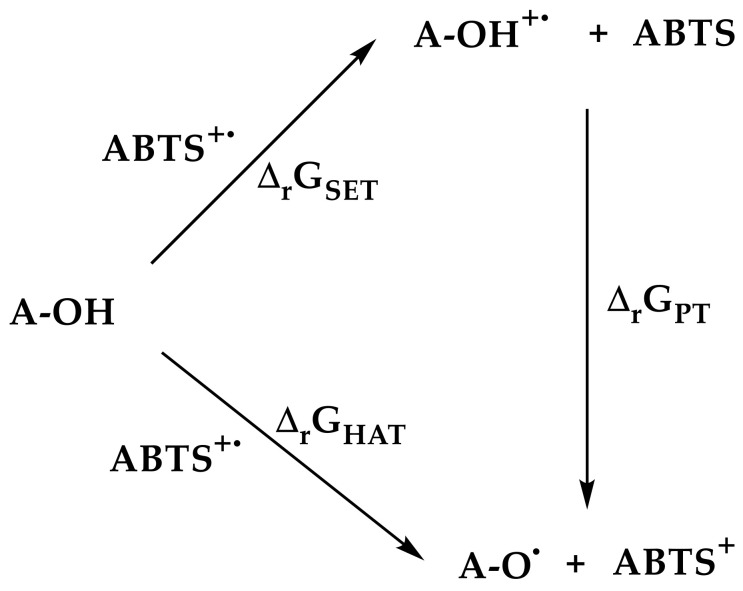
The investigation reaction mechanism: hydrogen atom transfer (hat) and single electron transfer followed by proton transfer (set-pt), between 2,2′-azino*bis*-(3-ethylbenzothiazoline-6-sulfonic acid) radical cation (**ABTS^•+^**) and **C-HB_1_**/**C-HB_2_** (**A-OH**).

In this case, an examination was conducted on the HAT and SET-PT mechanisms in order to elucidate the potential mechanism of antioxidant activity between the compounds under investigation and ABTS^•+^ (Figure 7).

The estimated values of thermodynamic parameters are presented in Table 6. The thermodynamic favorability of the transfer of a hydrogen atom from the –OH groups of **A-OH** (C-HB_1_/C-HB_2_) to ABTS^•+^ resulted in the formation of **A-O**^•^ and **ABTS^+^**. Like the discussion of reaction with the DPPH^•^, the reactivity of different positions of the **C-HB_1_** compound towards ABTS^•+^ decreases in the following order: **3-OH** > **2-OH** > **N2-H** > **4-OH**. On the other hand, the reactivity of different positions within the compound **C-HB_2_**, when subjected to a direct reaction with the ABTS radical decreases in the following order: **4-OH** > **3-OH/5-OH** > **N2-H**.

Both compounds exhibit significant exergonic values for the initial stage of the SET-PT mechanism. Based on the analysis of Δ_r_*G*_SET_ values, it can be concluded that compound **C-HB_1_** (−116 kJ mol^−1^) has a higher electron transfer capacity towards ABTS^•+^ in comparison to **C-HB_2_** (−110 kJ mol^−1^). Nevertheless, the significantly high endergonic Δ_r_*G*_PT_ values (>124 kJ mol^−1^) observed in the second step of the mechanism represent a constraining factor for the manifestation of antioxidant activity via the SET-PT mechanism. Based on the Δ_r_*G*_HAT_ values as well as the total Δ_r_*G*_SET_ + Δ_r_*G*_PT_ values, it can also be concluded in this case that the 3-OH positions of the **C-HB_1_** compound and the 4-OH of the **C-HB_2_** compound represent the dominant positions that react with ABTS^•+^. For about 10 kJ mol^−1^, the 4-OH position of the **C-HB_2_** compound shows a better thermodynamic favorability towards ABTS compared to the 3-OH position of **C-HB_1_**. This result demonstrates a correlation with parameters that were gained through the experiment. It can be inferred that the HAT and SET-PT mechanisms are thermodynamically favorable and competitive for the reduction of ABTS^•+^ radical by the **C-HB_1_** and **C-HB_2_**.

### 3.5. ADMET Analysis

The observed efficacy of compounds **C-HB_1_** and **C-HB_2_** in inactivating reactive radical species justifies the assumption of using these compounds in further studies with the aim of potential application as dietary supplements or pharmaceutical agents. Consequently, these compounds underwent ADMET analysis utilizing the biopharmaceutical online server ADMETlab 2.0. Commercially available and widely used antioxidants were taken as a standard for comparison (vitamin C and gallic acid).

The process of drug absorption refers to the transfer of the drug into the systemic circulation of the human body. In order for oral medicine or supplement to enter the systemic circulation, it must traverse intestinal cell membranes by passive diffusion, carrier-mediated transport, or active transport mechanisms. Caco-2 cell lines, derived from human colon adenocarcinoma, serve as a viable substitute for human intestinal epithelium in assessing drug permeability in vivo, owing to their comparable morphology and functionality. A compound with a Caco-2 value greater than −5.15 cm s^−1^ is regarded as possessing favorable permeability across epithelial cells [45,46,47,48]. All substances that have undergone testing, including the commercially utilized vitamin C, have permeability levels that are situated at the threshold of what is considered to be optimal. It is evident that both investigated compounds have a superior capacity to permeate the epithelium of Caco-2 cells in comparison to vitamin C, as indicated in Table 7.

The Madin–Darby canine kidney cell (MDCK) model was originally designed as an in vitro model to evaluate permeability in the field of biomedical research. This criterion epitomizes the optimal balance in assessing the efficacy of drug absorption within the human body. A compound is deemed to exhibit favorable permeability when its value exceeds 2 × 10^−6^ cm s^−1^ [48,49]. Based on this criterion, it can be concluded that all the compounds that were evaluated have favorable absorption characteristics within the human body, as indicated in Table 7.

The absorption of an oral medicine in the human intestine, known as human intestinal absorption (HIA), is a crucial requirement for its efficacy. The values in question span a numerical scale of 0 to 1, wherein molecules possessing a value within the range of 0–0.3 are said to have favorable intestinal absorption, surpassing the threshold of 30% [50]. The data presented in Table 7 clearly indicate that the process of intestinal absorption is less efficient for newly synthesized compounds compared to vitamin C and gallic acid.

The oral bioavailability (F) of any drug delivered orally is unquestionably one of the most crucial pharmacokinetic characteristics. This parameter serves as a measure of the effectiveness of drug delivery into the systemic circulation. The drug’s availability is denoted by *F*_20%_ and *F*_30%_ values, with the numbers representing the percentage of availability [51]. Compounds possessing a value ranging from 0 to 0.3 are regarded as having favorable oral bioavailability. It is interesting that the newly synthesized compounds, according to this parameter, show better oral bioavailability than vitamin C and gallic acid (Table 7).

The subsequent phase of the ADMET analysis involves examining the drug’s distribution within the body. This process is typically characterized by several key parameters, including the drug’s binding affinity to plasma proteins (referred to as plasma protein binding, PPS%), the proportion of the unbound drug in the blood plasma (known as fraction unbound, Fu), and the volume distribution of the drug (expressed as volume distribution, VD in L/kg) [52]. Binding to serum proteins and subsequent transport throughout the body represents a significant method for medication distribution. Nevertheless, the drug’s bioavailability may be compromised due to its strong affinity for these proteins. If the percentage of protein precipitation (PPS%) exceeds 90%, it is inferred that the investigation compound being evaluated exhibits a strong affinity for proteins and a limited therapeutic efficacy. Both recently synthesized compounds exhibit much higher values of PPS% values compared to commercially used vitamin C and gallic acid. The *Fu* parameter provides information regarding the equilibrium between a protein that is bound and unbound in the serum. If the value exceeds 5%, it is possible to consider the distribution as potentially favorable. Given the compound’s strong binding to serum proteins, it is reasonable to anticipate a moderate level of distribution through passive transport, as indicated by the Fu values outlined in Table 7.

Drug volume distribution represents a theoretical concept that pertains to the relationship between the administered dose of a drug and its actual concentration in the bloodstream. It serves as a crucial measure for characterizing the distribution of pharmaceuticals within the body. The distribution of the substance within the body is deemed satisfactory when the volume of distribution (VD) falls within the range of 0.04–20 L kg^−1^ [53]. Compounds **C-HB_1_** and **C-HB_2_** exhibit a favorable distribution pattern comparable to that of conventional vitamin C and gallic acid supplements, as evidenced by the data presented in Table 7.

The metabolism of the newly synthesized compounds is the next stage in the discussion of ADMET analysis. Three isoenzymes—1A2, 3A4, and 2C9—metabolized more than 80% of medicines and belong to the human cytochrome P450 family [54]. Interacting drugs with these isoenzymes can be either inhibitors (I) or substrates (S). It is clear that all investigated compounds have isoenzyme activity (values < 1) and are metabolized in the aforementioned cycles (Table 8).

Drug elimination (clearance, CL) is a critical pharmacokinetic parameter that, along with the volume of distribution, determines the half-life and consequently the frequency of medication administration. If the result is greater than 5 mL min^−1^ kg^−1^, the drugs are thought to be promptly eliminated from the body [55]. In this case, the values of the investigated compounds are much lower than the recommended values and significantly lower than reference compounds (Table 8).

The half-life of a drug (T_1/2_) is a composite concept that combines both the drug’s clearance impact and the volume of distribution. If the value is between 0.0 and 0.3, it is assumed that the chemicals are swiftly eliminated from the body [55]. All compounds, including reference compounds, are slowly eliminated from the body, according to the values in the table (Table 8).

The final step in the ADMET study is to evaluate the toxicity of the newly synthesized compounds. The voltage-gated potassium channel expressed by the hERG (the human *ether-à-go-go*-related gene) gene regulates the exchange of the ventricular action potential and resting potential during cardiac depolarization and repolarization [56]. Blocking hERG might result in palpitations, fainting, or even rapid death. The examined substances had no effect on cardiotoxicity because these values are in the range from 0.0 to 0.3 (Table 9).

Drug-induced damage to the liver is a major threat to patient safety. Negative liver effects in clinical trials frequently result in the expensive and late termination of drug development initiatives [57]. The examined compounds **C-HB_1_** and **C-HB_2_** do not exhibit human hepatotoxicity (H-HT) because their H-HT values are in the range 0.0–0.3 (Table 9).

The Ames (AMES) test is a popular method for determining if a drug can cause mutations in the DNA structure of the test organism. It is a test for determining the mutagenic potential of compounds. The test aims to assess the mutagenic capacity of a substance, which holds significant importance given the established correlation between mutagenicity and carcinogenicity [58]. The values presented in Table 9 are in the 0.0–0.3 range, indicating that the investigated compounds are not mutagenic.

Drug-induced respiratory toxicity (RT) is frequently underestimated in its prevalence due to the absence of distinct initial indications or symptoms, as observed with traditional pharmaceuticals, and its potential to cause substantial morbidity and mortality [59]. The investigation compounds that were subjected to testing exhibit respiratory toxicity levels that approach the limit, approximately 0.3, which is a significantly greater value than vitamin C, as seen in Table 9.

## 4. Conclusions

This paper describes a green synthesis of coumarin–hydroxybenzohydrazide derivatives utilizing vinegar and ethanol as a catalyst and solvent. This experimental procedure facilitates the separation of highly purified products with a modest yield, obviating the need for further purification steps. These compounds’ antioxidant ability was determined using the DPPH, ABTS, and FRAP tests. The study’s findings show that even at very low concentrations, these compounds have outstanding DPPH and ABTS radical scavenging capacities, as well as a substantial reduction potential as measured by the FRAP assay. The antioxidant activity shown by these compounds is comparable to, or perhaps superior to, that of well-known antioxidants such as gallic acid, NDGA, and trolox. Experimentally obtained results are in good correlation with the thermodynamic parameters that describe the antiradical potential of investigated compounds. According to the theoretical calculations, HAT is the favorable mechanistic pathway when it comes to DPPH^●^ inactivation, while ABTS^+●^ inactivation follows both HAT and SET-PT mechanistic pathways.

Moreover, the coumarin–hydroxybenzohydrazides exhibited favorable-to-moderate cytotoxic effects and showed significant potential in controlling the redox status in HCT-116 colorectal cancer cells. The cytotoxicity was achieved by the compounds’ prooxidative activity, which resulted in the production of oxidative stress in cancer cells. This was accomplished by raising the concentrations of O_2_˙^−^, as well as measuring the levels of MDA as an indication of lipid peroxidation and GSH levels. Because cancer cells have a disrupted redox equilibrium in comparison to healthy cells, manipulating reactive oxygen species (ROS) could be a promising path for cancer treatments including coumarins. Furthermore, ROS-associated signaling pathways could be manipulated and studied further in the future. According to the parameters obtained through ADMET analysis, investigated compounds show a good pharmacokinetic profile, comparable to that of vitamin C and gallic acid. Vitamin C and gallic acid are naturally occurring antioxidants used in various industries and with antioxidative potential similar to **C-HB_1_** and **C-HB_2_**. Due to their low potential toxicity and good pharmacokinetic properties, investigated compounds should be considered for further biological and medicinal investigations with potential use in pharmaceutical, food, and other industrial branches.

## Figures and Tables

**Table 1:** The results of the DPPH test for products **C-HB_1_**, **C-HB_2_** and reference compounds.

Compound	DPPH Scavenging Ability (%)						IC_50_ (µM)	SF
	25 µM		50 µM		100 µM			
	20 min	60 min	20 min	60 min	20 min	60 min		
**C–HB_1_**	82.5 ± 0.8	85.2 ± 1.0	86.9 ± 0.6	88.5 ± 0.7	89.1 ± 1.0	90.1 ± 0.6	6.4 ± 0.1	2.0
**C–HB_2_**	92.5 ± 1.2	93.0 ± 1.2	93.6 ± 0.5	93.8 ± 0.4	94.0 ± 0.8	94.1 ± 0.6	2.5 ± 0.1	5.0
**NDGA**	94.6 ± 0.7	94.6 ± 0.6	94.2 ± 0.7	94.2 ± 0.7	94.5 ± 0.2	94.1 ± 0.7	1.7 ± 0.1	7.4
**Gallic acid**	93.4 ± 0.8	93.5 ± 0.6	94.5 ± 0.9	94.3 ± 0.7	97.1± 1.1	97.0± 0.9	2.6 ± 0.2	4.8

**Table 2:** ABTS^•+^ scavenging activity of products **C-HB_1_**, **C-HB_2_** and corresponding referent compounds.

Compound	ABTS Radical Cation Scavenging Activity						
	Scavenging Ability (%)						IC_50_ (µM)
	1 µM	2 µM	3 µM	4 µM	6 µM	8 µM	
**C–HB_1_**	-	-	-	47.9 ± 1.7	55.3 ± 0.9	64.7 ± 1.4	4.5 ± 0.1
**C–HB_2_**	20.4 ± 1.8	46.7 ± 0.9	68.9 ± 0.3	-	-	-	2.0 ± 0.2
**Trolox**	-	-	-	38.8 ± 2.1	55.3 ± 2.2	71.9 ± 2.6	5.3 ± 0.1

**Table 3:** Ferric ion redox potential of **C-HB_1_**, **C-HB_2_** and corresponding referent compounds expressed through the absorbance 700 nm.

Compounds	Absorbance 700 nm
**C–HB_1_**	0.3979 ± 0.0092
**C–HB_2_**	0.7725 ± 0.0242
**Ascorbic acid**	0.1249 ± 0.0022

**Table 4:** Cytotoxic effects—IC_50_ values (μM) of coumarins on HCT-116 cell lines, after 24 and 72 h.

Compounds	IC_50_ (μM)	
	24 h	72 h
**C–HB_1_**	154.42 ± 0.12	104.86 ± 0.05
**C–HB_2_**	102.96 ± 0.06	54.49 ± 0.07

**Table 5:** Calculated thermodynamic parameters (in kJ mol^−1^) of antioxidant mechanisms between investigated compounds **C–HB_1_** and **C–HB_2_** and 2,2-diphednyl-1-picrylhydrazyl (DPPH^•^) radical at CPCM/M06-2X/6-311++G(d,p) level of theory in methanol.

Compounds	Position	Mechanisms		
		HAT	SET	PT
		Δ_r_*G*_HAT_	Δ_r_*G*_SET_	Δ_r_*G*_PT_
**C–HB_1_**	2-OH	13	159	−146
	3-OH	9		−150
	4-OH	31		−127
	N2-H	23		−136
**C–HB_2_**	3-OH/5-OH	19	165	−146
	4-OH	−1		−166
	N2-H	28		−137

**Table 6:** Calculated thermodynamic parameters (in kJ mol^−1^) of antioxidant mechanisms between investigated compounds **C–HB_1_** and **C–HB_2_** and 2,2′-azino*bis*-(3-ethylbenzothiazoline-6-sulfonic acid) radical cation (**ABTS^•+^**) at CPCM/M06-2X/6-311++G(d,p) level of theory in methanol.

Compounds	Position	Mechanisms		
		HAT	SET	PT
		Δ_r_*G*_HAT_	Δ_r_*G*_SET_	Δ_r_*G*_PT_
**C–HB_1_**	2-OH	29	−116	145
	3-OH	25		141
	4-OH	47		163
	N2-H	38		154
**C–HB_2_**	3-OH/5-OH	35	−110	144
	4-OH	15		124
	N2-H	44		153

**Table 7:** Absorption and distribution parameters of investigated compounds **C-HB_1_** and **C-HB_2_** and reference standards (vitamin C and gallic acid) evaluated within the context of ADMET analysis.

Compounds	Absorption					Distribution		
	Caco-2p (cm s^−1^)	MDCK (cm s^−1^)	HIA	F_20%_	F_30%_	PPS %	Fu %	VD (L kg^−1^)
**C–HB_1_**	−5.282	1.40 × 10^−6^	0.095	0.364	0.042	99.08	5.650	0.556
**C–HB_2_**	−5.853	7.20 × 10^−6^	0.103	0.578	0.085	97.90	6.270	0.551
**Vitamin C**	−5.917	1.40 × 10^−4^	0.069	0.918	0.987	63.23	36.58	0.937
**Gallic acid**	−5.728	5.10 × 10^−6^	0.085	0.964	0.995	53.49	33.59	0.446

**Table 8:** Metabolism and elimination parameters of investigated compounds **C-HB_1_** and **C-HB_2_** and referent standards (vitamin C and gallic acid) evaluated within the context of ADMET analysis.

Compounds	Metabolism; P450						Elimination	
	1A2		3A4		2C9		T_1/2_ h	CL mL min^−1^ kg^−1^
	I	S	I	S	I	S		
**C-HB_1_**	0.269	0.062	0.027	0.043	0.314	0.089	0.953	1.125
**C-HB_2_**	0.304	0.063	0.025	0.040	0.390	0.138	0.951	1.227
**Vitamin C**	0.013	0.052	0.010	0.057	0.007	0.230	0.928	9.964
**Gallic acid**	0.023	0.075	0.026	0.039	0.188	0.061	0.947	10.108

**Table 9:** Toxicology parameters of investigated compounds **C-HB_1_** and **C-HB_2_** and referent standards (vitamin C and gallic acid) evaluated within the context of ADMET analysis.

Compounds	hERG	H-HT	AMES	RT
**C-HB_1_**	0.006	0.195	0.094	0.240
**C-HB_2_**	0.007	0.196	0.045	0.303
**Vitamin C**	0.015	0.168	0.027	0.096
**Gallic acid**	0.017	0.443	0.053	0.381

## Data Availability

Data are contained within the article and Supplementary Materials.

## Supplementary Materials

The following supporting information can be downloaded at https://www.mdpi.com/article/10.3390/antiox12122070/s1. Figure S1. ^1^H NMR (200 MHz, top) and ^13^C NMR (50 MHz, bottom) spectra of **C-HB1** recorded in DMSO-*d*_6_; Figure S2. ^1^H NMR (200 MHz, top) and ^13^C NMR (50 MHz, bottom) spectra of **C-HB2** recorded in DMSO-*d*_6_; Figure S3. IR spectrum of C-HB_1_; Figure S4. IR spectrum of C-HB_2_; Figure S5. NBO spin density distribution values of formed radical species formed in the reaction between **C-HB_1_** (a,c) and **C-HB_2_** (b,d) with DPPH radical; Table S1. Experimental and theoretical chemical shifts (ppm) in ^1^H NMR spectra of newly synthesized compounds **C–HB_1_** and **C–HB_2_**; Table S2. Experimental and theoretical chemical shifts (ppm) in ^13^C NMR spectra of newly synthesized compounds **C–HB_1_** and **C–HB_2_**; Table S3. The results of the DPPH test for products C-HB1, C-HB2 and referent compounds. Values used for IC_50_ determination.
