# Supplementary material for: An Effective, Green Synthesis Procedure for Obtaining Coumarin–Hydroxybenzohydrazide Derivatives and Assessment of Their Antioxidant Activity and Redox Status
Source: Antioxidants (Basel). 2023 Dec 1;12(12):2070. doi: 10.3390/antiox12122070 (PMC10740980; doi:10.3390/antiox12122070)
Supplement: Supplementary file 1 [file antioxidants-12-02070-s001.zip › antioxidants-2727150-supplementary.pdf]

Supplementary Material

for

An effective, green synthesis procedure for obtaining new coumarin-hydroxybenzohydrazide derivatives and assessment of their antioxidant activity and redox status

Edina H. Avdović[1,*], Žiko Milanović[1], Dušica Simijonović[1], Marko Antonijević[1], Milena Milutinović[2], Danijela Nikodijević[2], Nenad Filipović[3,*], Zoran Marković[1,4], Radiša Vojinović[5]

[1]Department of Science, Institute for Information Technologies, University of Kragujevac, Jovana Cvijića bb, 34000 Kragujevac, Serbia; edina.avdovic@pmf.kg.ac.rs (E.A); ziko.milanovic@uni.kg.ac.rs (Ž.M); dusicachem@kg.ac.rs (D.S); mantonijevic@uni.kg.ac.rs (M.A);
[2]Department of Biology and Ecology, Faculty of Science, University of Kragujevac, Radoja Domanovića 12, 34000 Kragujevac, Serbia; milena.milutinovic@pmf.kg.ac.rs (M.M).; danijela.nikodijevic@pmf.kg.ac.rs (D.N)
[3]Faculty of Engineering, University of Kragujevac, Sestre Janjic 6, 34000 Kragujevac, Serbia; fica@kg.ac.rs (N.F);
[4]Department of Natural Science and Mathematics, State University of Novi Pazar, Novi Pazar, Serbia; zmarkovic@uni.kg.ac.rs (Z.M);
[5]Faculty of Medical Sciences, University of Kragujevac, Svetozara Markovića 69, 34000 Kragujevac, Serbia; rhvojinovic@gmail.com (R.V);

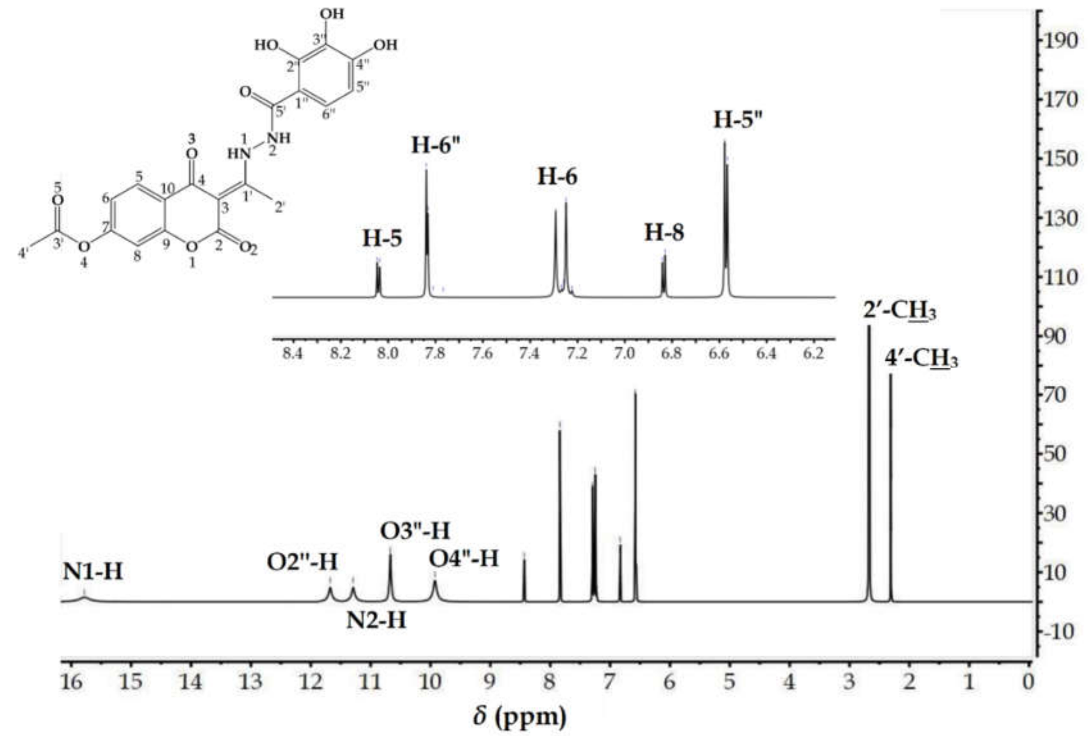

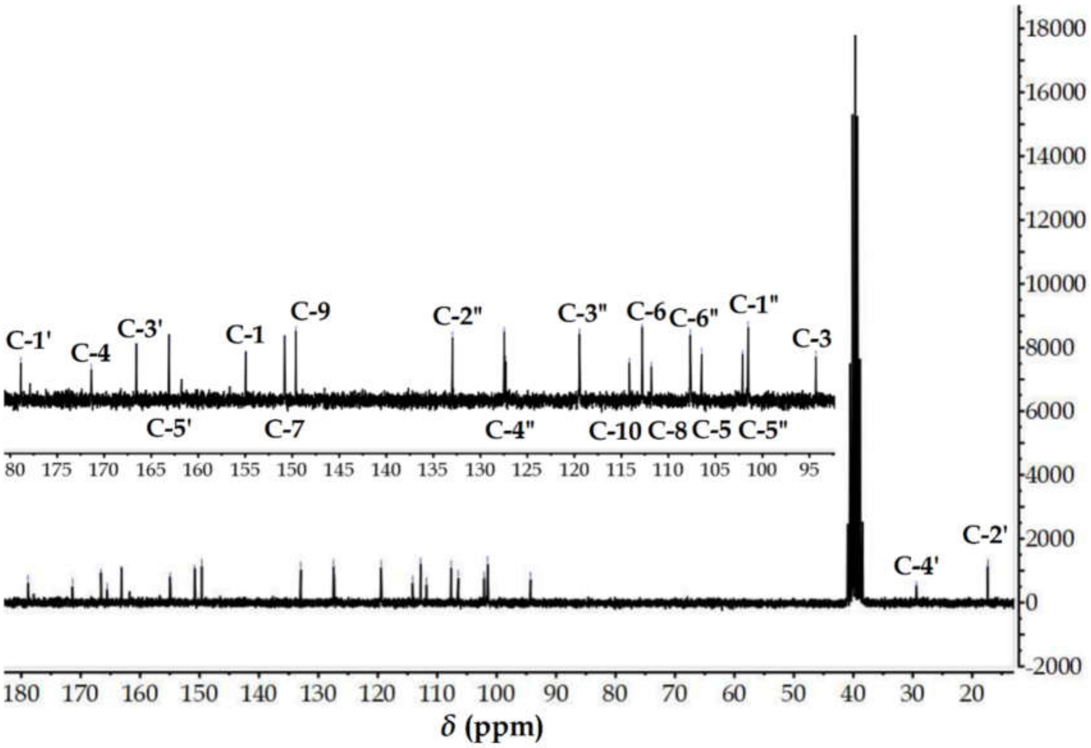

s

Figure S1. ^{1}H NMR (200 MHz, top) and ^{13}C NMR (50 MHz, bottom) spectra of **C-HB1** recorded in DMSO-d_6

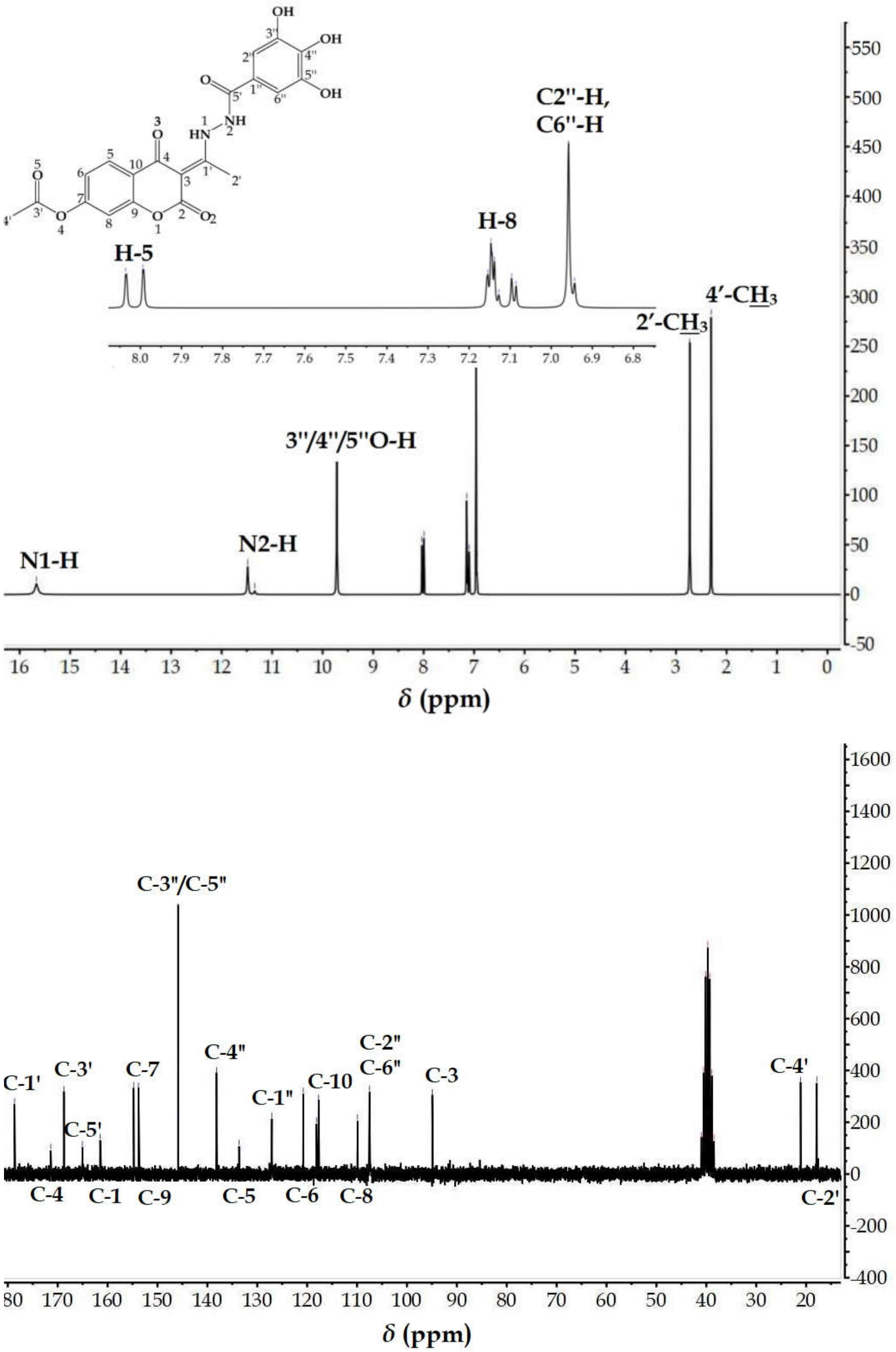

Figure S2. ^{1}H NMR (200 MHz, top) and ^{13}C NMR (50 MHz, bottom) spectra of **C-HB2** recorded in DMSO-d_6

Table S1. Experimental and theoretical chemical shifts (ppm) in ^{1}H NMR spectra of newly synthesized compounds **C–HB$_1$** and **C–HB$_2$**

Atoms	Experimental chemical shifts. δ (ppm)		Theoretical chemical shifts. δ (ppm)	
	**C–HB$_1$**	**C–HB$_2$**	**C–HB$_1$**	**C–HB$_2$**
**C4′–3H**	2.31	2.31	2.38	2.32
**C2′–3H**	2.67	2.85	2.70	2.79
**C5″–H**	6.57	/	6.76	/
**C2″–H**	/	6.95	/	7.23
**C8–H**	6.83	7.13	7.14	7.16
**C6–H**	7.27	7.13	7.22	7.22
**C6″–H**	7.84	6.95	7.27	7.18
**C5–H**	8.73	8.01	8.44	8.01
**O2″–H**	11.57	/	12.23	/
**O3″–H**	10.67		/	
**O4“–H**	9.93	9.87	/	/
**O5“–H**	/		/	
**N2–H**	11.29	11.48	/	/
**N1–H**	15.57	15.67	14.45	14.45
**MAE**	/	/	**1.4**	**0.3**
**R**	/	/	**0.993**	**0.995**

Table S2. Experimental and theoretical chemical shifts (ppm) in ^{13}C NMR spectra of newly synthesized compounds **C–HB$_1$** and **C–HB$_2$**

Atoms	Experimental chemical shifts, δ (ppm)		Theoretical chemical shifts, δ (ppm)	
	**C–HB$_1$**	**C–HB$_2$**	**C–HB$_1$**	**C–HB$_2$**
**C2′**	17.4	17.9	15.0	13.9
**C4′**	29.4	21.1	17.3	16.6
**C3**	98.7	94.9	98.7	97.6
**C1″**	102.1	120.8	103.2	122.9
**C5″**	106.5	149.5	107.0	145.7
**C8**	107.7	109.9	111.2	110.0
**C6″**	111.8	107.1	118.9	105.0
**C10**	112.8	117.7	119.0	118.3
**C6**	114.1	118.1	120.0	118.3
**C5**	119.5	127.1	129.2	129.0
**C3“**	127.3	145.9	132.6	143.9
**C4“**	127.5	138.2	151.5	137.0
**C2“**	133.0	107.1	151.9	105.2
**C9**	149.6	153.8	158.1	157.0
**C7**	150.8	154.8	160.7	158.9

**C1**	155.0	161.5	163.3	162.5
**C5′**	163.1	165.1	169.7	167.8
**C3′**	166.6	168.7	175.8	174.8
**C4**	171.4	171.4	183.2	182.1
**C1′**	178.9	178.7	186.0	184.7
**MAE**	/	/	**8.13**	**2.85**
**R**	/	/	**0.993**	**0.998**

Table S3. The results of the DPPH test for products C-HB1, C-HB2 and referent compounds. Values used for IC_{50} determination.

Compound	DPPH scavenging ability (%)									
	1 (μM)	1.5 (μM)	2 (μM)	2.5 (μM)	3 (μM)	6 (μM)	6.5 (μM)	7 (μM)	IC_{50} (μM)	SF
**C–HB$_1$**	/	/	/	/	/	49.2	49.9	55.4	6.4 ± 0.1	2.0
**C–HB$_2$**	34.4	35.8	45.6	51.3	63.1	/	/	/	2.5 ± 0.1	5.0
**NDGA**	40.2	42.2	56.3	59.9	79.52	/	/	/	1.7±0.1	7.4
**Quercetin**	30.7	45.1	53.9	60.9	77.6	/	/	/	1.9±0.1	6.6

C-HB$_1$

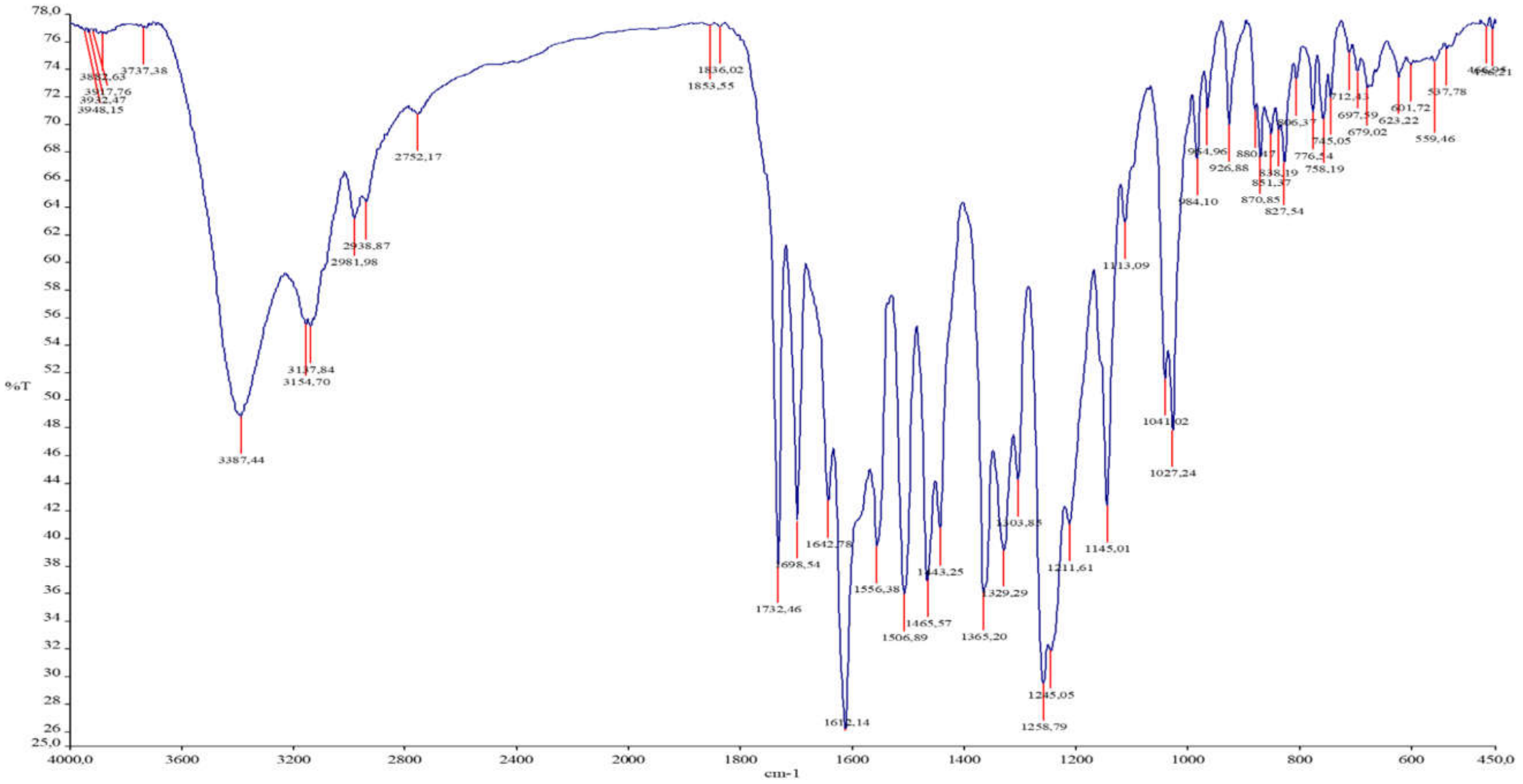

Figure S3. IR spectrum of C-HB$_1$

C-HB$_2$

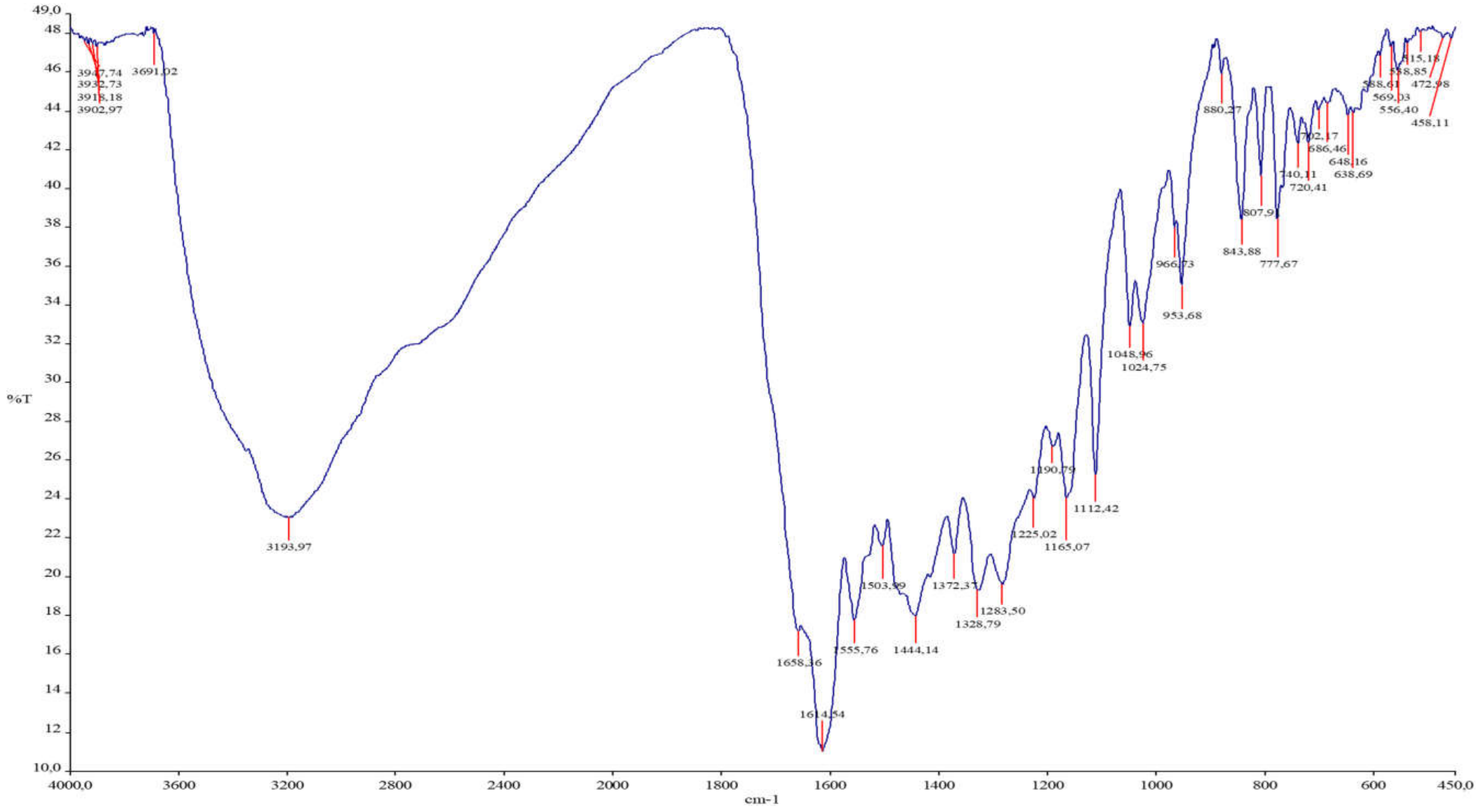

Figure S4. IR spectrum of C-HB$_2$

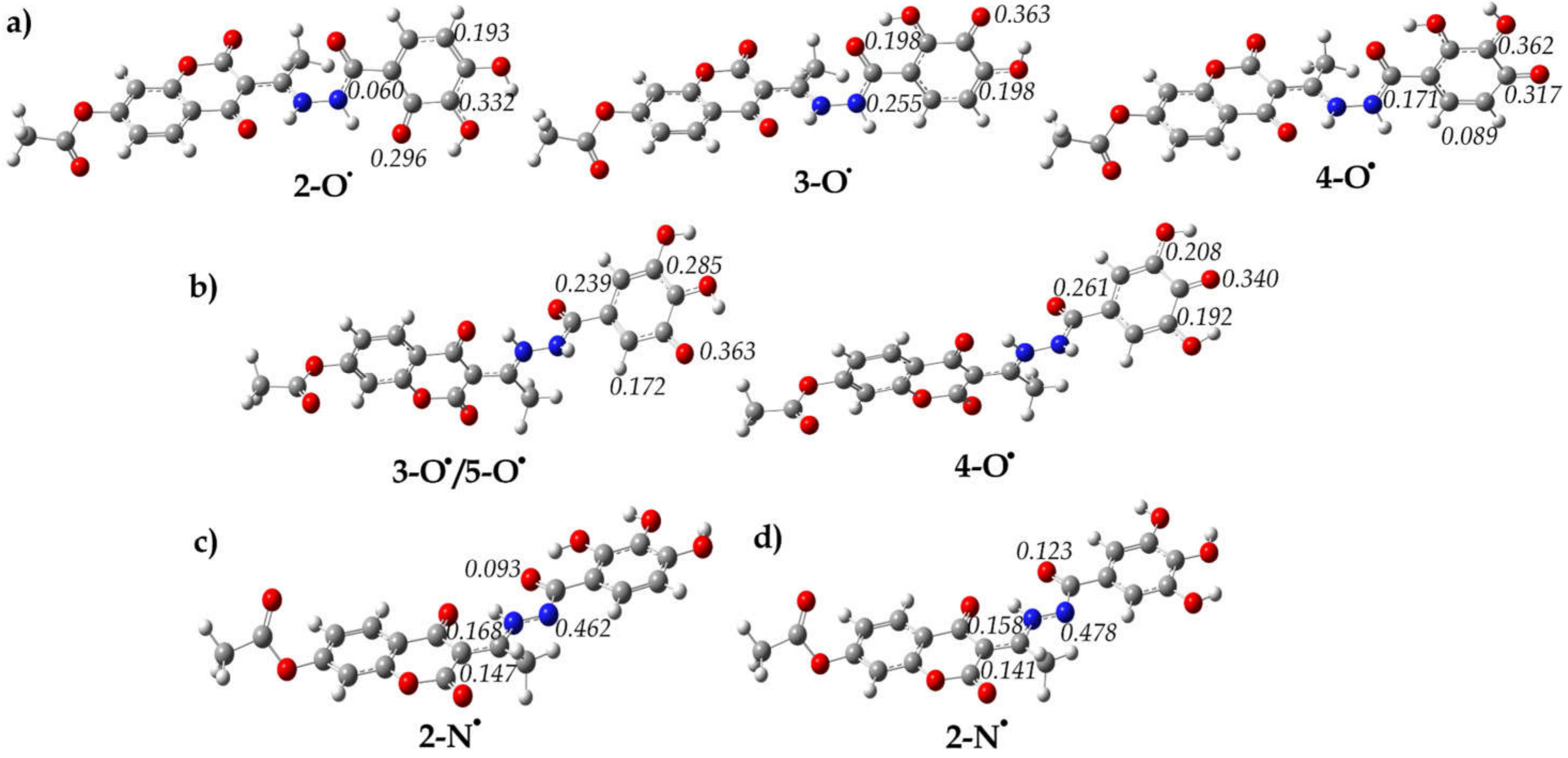

Figure S5. NBO spin density distribution values of formed radical species formed in the reaction between **C-HB₁** (a,c) and **C-HB₂** (b,d) with DPPH radical.

C–HB$_1$

0 1	x	y	z
C	-1.79323800	2.10106200	0.08358600
C	-3.76832700	0.73274200	0.25671500
C	-3.10036200	-0.42243800	-0.12533700
C	-1.66623800	-0.35918600	-0.45210200
C	-1.03186900	0.94034300	-0.35332900
H	-5.63656100	1.62236500	0.86672500
C	-5.12550700	0.71642500	0.56839800
C	-3.80992900	-1.62718100	-0.19343300
C	-5.15417000	-1.67302700	0.11590500
C	-5.79238000	-0.48981600	0.49331100
H	-3.27596200	-2.52121000	-0.49005400
H	-5.71362600	-2.59845500	0.07383600
O	-3.13623900	1.93230100	0.34932800
O	-1.38356800	3.22399800	0.25140700
O	-1.07484900	-1.39404000	-0.79254200
C	0.35000300	1.09833800	-0.64035500
C	1.07032100	2.40542000	-0.57746800
H	0.51414800	3.15516800	-1.13610400
H	1.10983800	2.74771500	0.45769800
H	2.07662900	2.30957200	-0.97376300
O	-7.12317500	-0.50750800	0.87824700
C	-8.06869200	-0.80251100	-0.06058100
C	-9.43054500	-0.82143500	0.55752800
H	-9.61732800	0.12993200	1.05649500
H	-10.17677000	-0.99945800	-0.21155300
H	-9.47090100	-1.60900500	1.31158400
O	-7.79736600	-1.00480900	-1.20998100
N	1.04093100	0.02074200	-0.99567800
H	0.54003800	-0.87824900	-0.99982800
N	2.40824500	0.02026100	-1.15103600

C	3.20064900	0.00692000	-0.02991300
H	2.73467600	-0.34710900	-2.03310600
C	4.64888300	-0.17470800	-0.22957000
C	5.27011100	-0.16799900	-1.48872200
C	5.44515100	-0.37234300	0.91334600
C	6.62861600	-0.37323800	-1.61920500
H	4.69675500	0.02670200	-2.38604100
C	6.81448200	-0.58852600	0.77800500
C	7.40524600	-0.59214900	-0.47904300
O	2.69735400	0.17260300	1.07892700
O	7.61916700	-0.79513500	1.85551800
H	7.09292500	-0.73715600	2.66373400
O	8.73379700	-0.79546600	-0.60474300
H	9.12185900	-0.92170400	0.27161400
H	7.11019700	-0.36003200	-2.58800300
O	4.98537600	-0.37867800	2.17948000
H	4.03264500	-0.15238200	2.15221500

C–HB$_1$ – Radical (2-O)

0 2	x	y	z
C	-1.79725800	1.99923200	-0.54333200
C	-3.78650000	0.76214300	0.01905600
C	-3.08863900	-0.41731700	0.23796400
C	-1.62805200	-0.44456700	0.04594500
C	-1.00384100	0.79110500	-0.38024100
H	-5.70097200	1.75516100	0.00577900
C	-5.16853700	0.82904100	0.17836200
C	-3.79352200	-1.56122700	0.63053300
C	-5.16315500	-1.52169100	0.80009600
C	-5.83003900	-0.31803900	0.56727800
H	-3.23772500	-2.47487400	0.80011700
H	-5.72223000	-2.39663800	1.10646000

O	-3.16136900	1.90896400	-0.35489000
O	-1.40097500	3.10430600	-0.82585700
O	-1.01026800	-1.50067700	0.24575600
C	0.39858300	0.85731100	-0.60987400
C	1.10362500	2.07965800	-1.09996200
H	0.56719300	2.49596800	-1.94953500
H	1.10025300	2.83727300	-0.31492600
H	2.12718900	1.84497000	-1.37708900
O	-7.19726800	-0.23707100	0.78607000
C	-8.02500700	-0.81445900	-0.13158400
C	-9.45407800	-0.66631700	0.28399100
H	-9.68887900	0.39226600	0.40102000
H	-10.09992100	-1.11608800	-0.46462200
H	-9.60023100	-1.15024300	1.25062200
O	-7.61508300	-1.35002000	-1.12232800
N	1.12016700	-0.23479200	-0.39504600
H	0.62449100	-1.06496200	-0.04537300
N	2.49288800	-0.26361800	-0.45550100
C	3.22633300	0.33884400	0.53251800
H	2.92656100	-0.92885100	-1.09000000
C	4.69673100	0.10080300	0.47033600
C	5.49003500	0.75620700	1.37511400
C	5.32330700	-0.79079600	-0.48290400
C	6.89885900	0.60576400	1.42517700
H	5.01354900	1.42134200	2.08524500
C	6.77515200	-0.92414700	-0.40196800
C	7.54706700	-0.22896900	0.54388900
O	2.70959900	1.04835900	1.37573500
O	7.37867100	-1.72980800	-1.25428500
H	6.69844100	-2.12567500	-1.83086000
O	8.88433400	-0.37713100	0.58593000
H	9.17399600	-0.99530100	-0.09769900

H	7.47795400	1.14903000	2.16073300
O	4.75268100	-1.46066800	-1.36718100

C–HB$_1$ – Radical (3-O)

0 2	x	y	z
C	-1.73763600	2.04345100	-0.13732100
C	-3.74687800	0.75357000	0.17918700
C	-3.09498700	-0.45889000	0.00305100
C	-1.65165400	-0.47303900	-0.28809000
C	-0.99641500	0.81659300	-0.39402500
H	-5.60967900	1.76418600	0.57866000
C	-5.11335100	0.81256100	0.44077700
C	-3.83159000	-1.64583800	0.09671000
C	-5.18609900	-1.61682300	0.35991300
C	-5.80752800	-0.37739300	0.52682100
H	-3.31155000	-2.58545100	-0.04130000
H	-5.76658300	-2.52693200	0.43711000
O	-3.08981300	1.94143200	0.11225200
O	-1.30159300	3.16831400	-0.10886900
O	-1.07219100	-1.55771200	-0.43818300
C	0.38333500	0.90641400	-0.70892500
C	1.11498800	2.19616100	-0.89001600
H	0.53167500	2.86215200	-1.52113600
H	1.22419800	2.68807200	0.07821400
H	2.09456200	2.02531500	-1.32709800
O	-7.15106000	-0.31158300	0.85821300
C	-8.06773200	-0.72800500	-0.06315100
C	-9.45290300	-0.61907800	0.49066200
H	-9.66673400	0.42662600	0.71711700
H	-10.16574800	-0.99837400	-0.23602500
H	-9.51889000	-1.18361800	1.42116200
O	-7.75757900	-1.10791000	-1.15629100

N	1.06428800	-0.22475900	-0.86982500
H	0.56014000	-1.10759300	-0.71311100
N	2.42811800	-0.26085100	-1.04372300
C	3.24157200	-0.05192500	0.03539600
H	2.74288300	-0.74345100	-1.87266300
C	4.69496500	-0.23086500	-0.15492800
C	5.29452000	-0.43078200	-1.41517200
C	5.50678700	-0.18807900	0.98575000
C	6.65930700	-0.62347200	-1.58256000
H	4.68501500	-0.41388700	-2.30996800
C	6.94342800	-0.40104300	0.86777900
C	7.47545100	-0.62090100	-0.46719500
O	2.76207500	0.30020900	1.11156300
O	7.71547600	-0.39363200	1.84377700
O	8.78493100	-0.80786600	-0.56019500
H	9.15218400	-0.76099400	0.34001400
H	7.08608600	-0.76796100	-2.56593300
O	5.06640000	0.03568500	2.20744800
H	4.09694700	0.20997900	2.14408900

C–HB$_1$ – Radical (4-O)

0 2	x	y	z
C	-1.74587600	2.08851000	-0.01780000
C	-3.72807700	0.74085100	0.22234300
C	-3.06305500	-0.43824500	-0.08477200
C	-1.62656400	-0.40086900	-0.40395300
C	-0.98843200	0.90103700	-0.38557400
H	-5.59597900	1.67446500	0.76305200
C	-5.08766600	0.74986500	0.52322200
C	-3.77846700	-1.64148400	-0.08727000
C	-5.12546900	-1.66203200	0.21235800
C	-5.76041900	-0.45539800	0.51384900

H	-3.24712400	-2.55393300	-0.32679400
H	-5.68988800	-2.58536600	0.21906600
O	-3.09086200	1.94111100	0.24913700
O	-1.32990400	3.21611000	0.09163700
O	-1.03616700	-1.45663300	-0.67378300
C	0.39238900	1.03675700	-0.68251200
C	1.11416400	2.34406100	-0.71507100
H	0.54600600	3.05907800	-1.30581800
H	1.17826600	2.74753000	0.29668100
H	2.11148900	2.22335200	-1.12765500
O	-7.09470300	-0.44408200	0.88656100
C	-8.03272500	-0.78566300	-0.04425900
C	-9.40185400	-0.75251400	0.55679300
H	-9.60988300	0.25365600	0.92331900
H	-10.13463900	-1.03938800	-0.19199100
H	-9.44076500	-1.43332100	1.40788300
O	-7.75023300	-1.05680800	-1.17670400
N	1.08200000	-0.06540400	-0.96075200
H	0.57970300	-0.96238900	-0.90490200
N	2.44914900	-0.07903200	-1.11503500
C	3.23947000	0.00605500	0.00099000
H	2.77503000	-0.50323000	-1.97134700
C	4.69185600	-0.18891500	-0.17895600
C	5.30836100	-0.28573200	-1.47539600
C	5.46927900	-0.28779100	0.96975500
C	6.63353200	-0.50885000	-1.64721700
H	4.69823700	-0.14852800	-2.35940200
C	6.85706400	-0.53349200	0.81721600
C	7.48825000	-0.65893100	-0.49505700
O	2.73929100	0.26336900	1.09308100
O	7.61866900	-0.65357300	1.88080200
H	8.52725200	-0.81847600	1.56204400

O	8.71040900	-0.87493100	-0.51902100
H	7.08167200	-0.57094200	-2.62991300
O	5.02142500	-0.18063600	2.21995600
H	4.06462400	0.03380900	2.17083200

C–HB$_1$ – Radical (N)

0 2	x	y	z
C	2.07607200	2.50924500	-0.05718700
C	3.85600100	0.89884500	-0.20177300
C	2.99270800	-0.18535300	-0.28244500
C	1.54052900	0.02837200	-0.23680000
C	1.08591600	1.42074800	-0.09603700
H	5.89879600	1.58541300	-0.17150200
C	5.23642500	0.73213700	-0.23335000
C	3.53005700	-1.47396500	-0.40435700
C	4.89489200	-1.66735600	-0.44381800
C	5.73069000	-0.55158200	-0.35413000
H	2.84662200	-2.31115400	-0.47011000
H	5.32295200	-2.65619000	-0.54367600
O	3.40552200	2.17906700	-0.09341600
O	1.83528300	3.68578600	0.00422200
O	0.77352000	-0.93140100	-0.30844300
C	-0.26729400	1.75481500	0.00386700
C	-0.80821700	3.13683800	0.17685500
H	-0.34720400	3.60729100	1.04455400
H	-0.54041300	3.74455100	-0.68831700
H	-1.88613900	3.10589700	0.29141300
O	7.10230300	-0.69889500	-0.45366700
C	7.75787400	-1.37103000	0.53915100
C	9.21768800	-1.46552200	0.22963400
H	9.62978900	-0.46146300	0.12101500
H	9.72502600	-1.99693800	1.02975600

H	9.35299300	-1.98689200	-0.71876800
O	7.19702500	-1.79751000	1.50752600
N	-1.17309400	0.73991900	-0.05632900
H	-0.80211600	-0.22229100	-0.17471500
N	-2.46233200	0.93244100	0.02870300
C	-3.15767400	-0.27943200	-0.06081100
C	-4.60823700	-0.15603900	0.03173900
C	-5.25118300	1.08582200	0.19162300
C	-5.39203600	-1.32558400	-0.04070600
C	-6.62363500	1.16914900	0.27730300
H	-4.64822400	1.98189500	0.24561300
C	-6.77713700	-1.23701500	0.04618900
C	-7.39141600	0.00114200	0.20490200
O	-2.57551000	-1.35901500	-0.20462800
O	-7.57887800	-2.33467900	-0.01771300
H	-7.03457500	-3.12406500	-0.13437400
O	-8.73370300	0.08245100	0.28991900
H	-9.11362100	-0.80444300	0.22629300
H	-7.12763400	2.11874400	0.40089500
O	-4.89556700	-2.56727000	-0.19377100
H	-3.92035200	-2.50056100	-0.24402400

C–HB$_1$ – Anion (2-O)

-1 1	x	y	z
C	2.07548107	-0.62104938	-1.01114276
C	3.52151039	1.25074292	-1.46133292
C	2.46786293	2.11428414	-1.73247522
C	1.08593733	1.61716554	-1.66866149
C	0.91668692	0.21817496	-1.31679433
H	5.65524707	1.00733791	-1.31057795
C	4.84166108	1.68656102	-1.52663408
C	2.74603521	3.44285992	-2.07063759

C	4.04575665	3.90090562	-2.13888207
C	5.08200549	3.00369096	-1.86617953
H	1.90865369	4.09808193	-2.27640205
H	4.26864633	4.92343935	-2.40821874
O	3.32729278	-0.04835467	-1.12088627
O	2.06123225	-1.77166020	-0.66875304
O	0.14704114	2.38652543	-1.91362139
C	-0.37266688	-0.34577689	-1.21945369
C	-0.63827187	-1.76999142	-0.85302109
H	-0.02034265	-2.42907373	-1.45929363
H	-0.33891423	-1.93427261	0.18335833
H	-1.69058673	-2.01023889	-0.97554444
O	6.40607015	3.40602417	-1.84474494
C	6.92887088	4.09305765	-2.90515462
C	8.36518596	4.43027422	-2.63292867
H	8.92317660	3.51653248	-2.42594609
H	8.78162897	4.94356876	-3.49521566
H	8.42669896	5.06428611	-1.74721324
O	6.30874568	4.36347344	-3.88785286
N	-1.41802800	0.44387336	-1.48477791
H	-1.19741203	1.44051755	-1.65430133
N	-2.72090232	0.06315501	-1.25301029
C	-3.19188200	0.02204200	0.04209700
H	-3.35077526	0.21527057	-2.02466653
C	-4.63675200	-0.19967000	0.22362900
C	-5.27111400	-0.27297900	1.47485600
C	-5.41972800	-0.35149800	-0.93473300
C	-6.62553900	-0.51074300	1.58302200
H	-4.71040000	-0.10998700	2.38709300
C	-6.78506300	-0.59991400	-0.82167000
C	-7.38883600	-0.68263900	0.42553000
O	-2.41081630	0.13612936	0.97801458

O	-7.57385400	-0.76021600	-1.91865800
H	-7.03046900	-0.65273100	-2.70940300
O	-8.71146200	-0.91610900	0.52403200
H	-9.08074100	-0.99721900	-0.36452000
H	-7.12029500	-0.55963400	2.54371500
O	-4.95507100	-0.28117200	-2.19537900

C–HB$_1$ – Anion (3-O)

-1 1	x	y	z
C	-1.76169100	2.10005600	0.07054900
C	-3.73488400	0.72912200	0.25069400
C	-3.06015800	-0.42885800	-0.11044000
C	-1.62364300	-0.36436200	-0.42947500
C	-0.99663500	0.93802000	-0.35074200
H	-5.61057200	1.62002500	0.83625800
C	-5.09423100	0.71199900	0.55380800
C	-3.76477100	-1.63694500	-0.16595700
C	-5.11106900	-1.68355400	0.13512800
C	-5.75595000	-0.49771300	0.49173000
H	-3.22547100	-2.53289500	-0.44666100
H	-5.66709500	-2.61145600	0.10221200
O	-3.10717700	1.93134100	0.33090800
O	-1.35688600	3.22635200	0.23128400
O	-1.02670200	-1.40350000	-0.74899800
C	0.38836700	1.09783300	-0.63990000
C	1.09768000	2.41230400	-0.61386000
H	0.52431100	3.14862900	-1.17247900
H	1.15908600	2.76937600	0.41521900
H	2.09655000	2.31365700	-1.02854100
O	-7.08969800	-0.51588500	0.86834200
C	-8.02793600	-0.81500100	-0.07581300
C	-9.39442900	-0.83231600	0.53226900

H	-9.58437400	0.11959000	1.02898500
H	-10.13510900	-1.01085600	-0.24203200
H	-9.44084500	-1.61913200	1.28677700
O	-7.74853300	-1.02215900	-1.22253300
N	1.08763000	0.01955600	-0.96270300
H	0.59269800	-0.88205400	-0.94512600
N	2.45561400	0.02800200	-1.12037000
C	3.24725000	0.06650000	0.01632100
H	2.77723100	-0.45870000	-1.94439800
C	4.68771100	-0.14383900	-0.17467200
C	5.26802500	-0.15124300	-1.45794700
C	5.49336200	-0.36053400	0.96697300
C	6.62335200	-0.39605100	-1.60535100
H	4.68007900	0.07817800	-2.33714400
C	6.87938700	-0.63344800	0.84880900
C	7.39280200	-0.64061000	-0.47429900
O	2.71882900	0.29921600	1.10222400
O	7.68514300	-0.86199300	1.83020600
O	8.72491800	-0.89295700	-0.56553300
H	8.98856900	-1.01578900	0.36790900
H	7.09126300	-0.39104000	-2.58266900
O	4.99675300	-0.33792700	2.23186100
H	4.06255600	-0.06849800	2.17414400

C–HB$_1$ – Anion (4-O)

-1 1	x	y	z
C	-1.75341200	2.08160900	-0.06175000
C	-3.72736100	0.72790500	0.21699000
C	-3.05867700	-0.45008400	-0.08591100
C	-1.62455500	-0.40680900	-0.42123600
C	-0.99573000	0.89625600	-0.42528800

H	-5.59438700	1.65592600	0.77118900
C	-5.08371000	0.73187100	0.53406300
C	-3.76697900	-1.65709400	-0.06855600
C	-5.11072700	-1.68264700	0.24688200
C	-5.74882400	-0.47728300	0.54476400
H	-3.23287100	-2.56857700	-0.30565000
H	-5.67003300	-2.60899100	0.26938500
O	-3.09661600	1.93075000	0.22374500
O	-1.34579600	3.21474200	0.03168200
O	-1.03258800	-1.46450100	-0.68500000
C	0.38765300	1.03633800	-0.73915200
C	1.09486500	2.35073600	-0.80483400
H	0.51206900	3.04936500	-1.40097000
H	1.16959600	2.77170600	0.19876000
H	2.08826700	2.22517300	-1.22537200
O	-7.08007800	-0.47353200	0.93261500
C	-8.02688800	-0.77042100	-0.00311200
C	-9.39142100	-0.75444200	0.60951300
H	-9.56451300	0.21039800	1.08722500
H	-10.13751400	-0.93649500	-0.15876000
H	-9.44796200	-1.52475000	1.38013500
O	-7.75511500	-0.99979700	-1.14759600
N	1.08463500	-0.05791200	-1.00125000
H	0.59190800	-0.95672000	-0.92219900
N	2.45188300	-0.05191700	-1.17828800
C	3.24950700	0.02169400	-0.03368500
H	2.75125900	-0.62716400	-1.95212200
C	4.66881000	-0.21029800	-0.19592500
C	5.28303700	-0.40677600	-1.45180100
C	5.49774300	-0.24482600	0.96776300
C	6.63101800	-0.64413600	-1.57785200
H	4.69291700	-0.34406700	-2.35922400

C	6.84457000	-0.49213600	0.83849500
C	7.47747500	-0.70594200	-0.42762100
O	2.70877500	0.32227200	1.03652100
O	7.65933800	-0.54090100	1.93630700
H	8.53531200	-0.72763400	1.55499000
O	8.73015100	-0.92431700	-0.44586800
H	7.08117000	-0.78115900	-2.55429100
O	5.00409400	-0.05033200	2.21596300
H	4.05344400	0.15562300	2.11624600

C–HB$_1$ – Anion (N)

-1 1

C	2.09250900	2.46439700	0.12141300
C	3.88007900	0.87245800	-0.15590300
C	3.00382200	-0.20127800	-0.23091600
C	1.54853300	0.03666300	-0.14032600
C	1.12312400	1.40449900	-0.02650000
H	5.93202400	1.54037500	-0.19274400
C	5.25926400	0.69466900	-0.25165500
C	3.52438400	-1.48844400	-0.40617300
C	4.88677400	-1.69324300	-0.50873000
C	5.73500100	-0.58842600	-0.42822200
H	2.82993900	-2.31732100	-0.46443800
H	5.30331600	-2.68259400	-0.64940900
O	3.43827300	2.14186300	0.02330000
O	1.88218500	3.63876200	0.33624200
O	0.77609000	-0.93742300	-0.18180900
C	-0.28154400	1.74725700	-0.01946900
C	-0.78291800	3.15865600	-0.06944400
H	-0.63270800	3.63838200	0.89973200
H	-0.21232300	3.73254600	-0.79614300

H	-1.84000300	3.15993600	-0.31553700
O	7.10448100	-0.75652900	-0.58926800
C	7.80162600	-1.33693400	0.42676700
C	9.24815600	-1.46515400	0.06598800
H	9.66798800	-0.47041400	-0.09126500
H	9.77878100	-1.97286400	0.86653500
H	9.34360200	-2.02037300	-0.86753600
O	7.28687400	-1.67365500	1.45584100
N	-1.17031400	0.78592000	0.00420000
H	-0.82994700	-0.18623400	0.00039300
N	-2.52805800	0.99399400	0.00863700
C	-3.17384100	-0.15885700	0.01100800
C	-4.66093000	-0.08440300	0.02394200
C	-5.36834800	1.12352400	0.02546100
C	-5.38632100	-1.28591800	0.03497500
C	-6.75301600	1.14850600	0.03805600
H	-4.80790700	2.04948000	0.01649600
C	-6.78087800	-1.26163100	0.04813100
C	-7.46297400	-0.05346900	0.04982800
O	-2.62073500	-1.30932100	0.00565800
O	-7.51329700	-2.41479800	0.06031700
H	-6.90327200	-3.16410900	0.05792000
O	-8.82221500	-0.03604700	0.06314100
H	-9.14623500	-0.94575300	0.07172800
H	-7.30314800	2.08104000	0.03934200
O	-4.80564000	-2.50298500	0.03444600
H	-3.81463100	-2.29766200	0.02274900

C–HB_2

0 1	x	y	z
C	-1.89219700	2.00713200	0.38216000
C	-3.88969500	0.66095900	0.36330200

C	-3.20782700	-0.48898000	-0.01109100
C	-1.75346600	-0.43086700	-0.23761000
C	-1.11875100	0.85941800	-0.06500000
H	-5.78881900	1.55516800	0.86084900
C	-5.26687600	0.65275900	0.57046700
C	-3.92313800	-1.68030500	-0.17696600
C	-5.28817000	-1.71731800	0.02649100
C	-5.93943200	-0.53989000	0.39665300
H	-3.37868500	-2.57027300	-0.46673900
H	-5.85490000	-2.63143200	-0.09530800
O	-3.25233900	1.84650700	0.54997700
O	-1.48054800	3.11288500	0.63777100
O	-1.14894400	-1.46152600	-0.56806600
C	0.27567500	1.02054900	-0.29132600
C	0.99218900	2.32700100	-0.18357200
H	0.43227100	3.09870200	-0.70618900
H	1.04347500	2.62400300	0.86545200
H	1.99612100	2.24566500	-0.59008500
O	-7.30138500	-0.56065500	0.65800700
C	-8.15212900	-0.64912600	-0.40476500
C	-9.57239200	-0.69599600	0.06185100
H	-9.79359900	0.20336000	0.63801500
H	-10.23502000	-0.76566500	-0.79606300
H	-9.70911900	-1.55620300	0.71831100
O	-7.76593800	-0.67554500	-1.53898700
N	0.98216900	-0.05030400	-0.63038900
H	0.48345400	-0.94880800	-0.67143900
N	2.35283100	-0.04224600	-0.74916500
C	3.12327700	-0.02437800	0.39643300
H	2.70706100	-0.44842700	-1.60416300
C	4.58801900	-0.17273900	0.16905500
C	5.34272100	-0.66959500	1.23221500

C	5.19063400	0.17878000	-1.03822800
C	6.70864600	-0.83567700	1.07091700
H	4.85933000	-0.92717200	2.16697100
C	6.56236600	0.01705000	-1.18479400
H	4.63554800	0.61448900	-1.85976400
C	7.32106600	-0.49445900	-0.13479000
O	2.62495500	0.13348700	1.49195800
O	7.16129100	0.37033800	-2.34853700
H	8.11088100	0.20758300	-2.27873100
O	8.65532900	-0.63372000	-0.33514700
H	9.07156900	-0.96660800	0.47073800
O	7.55569900	-1.32080600	2.01811600
H	7.08734800	-1.52014500	2.83586800

C–HB_2 – Radical (3-O/5-O)

0 2	x	y	z
C	1.86156300	2.01940700	-0.39065200
C	3.86159200	0.67778700	-0.35920000
C	3.17584900	-0.48216100	-0.02485600
C	1.71715800	-0.43345400	0.17176700
C	1.08251100	0.86027100	0.01759200
H	5.76808100	1.58895400	-0.79321300
C	5.24313400	0.67879900	-0.53441000
C	3.89193500	-1.67447000	0.13145000
C	5.26124500	-1.70247100	-0.04129600
C	5.91654400	-0.51475400	-0.36998500
H	3.34467700	-2.57228000	0.38993600
H	5.82805000	-2.61733700	0.07395400
O	3.22442700	1.86523700	-0.53596600
O	1.45079200	3.12878300	-0.63029100
O	1.10830300	-1.47217000	0.46529900

C	-0.31282900	1.01576700	0.23021600
C	-1.02823200	2.32457600	0.14679100
H	-0.47602400	3.08145400	0.69871800
H	-1.06155000	2.65169700	-0.89394200
H	-2.03823900	2.23473400	0.53581400
O	7.28383300	-0.52177400	-0.59998900
C	8.11220900	-0.67538500	0.47351500
C	9.54207500	-0.68695400	0.03484700
H	9.78095400	0.27025000	-0.43104700
H	10.18493300	-0.85642800	0.89391100
H	9.68844100	-1.46823900	-0.71155000
O	7.70286700	-0.77203700	1.59563600
N	-1.02333500	-0.06477000	0.53435900
H	-0.52578700	-0.96469900	0.56041000
N	-2.39401100	-0.05757500	0.65270000
C	-3.16388700	-0.04428200	-0.48857200
H	-2.74919000	-0.44012300	1.51879400
C	-4.63457200	-0.20041600	-0.25295800
C	-5.37176100	-0.75819600	-1.25879700
C	-5.23448600	0.22609300	0.96729100
C	-6.78877000	-0.94980800	-1.08280900
H	-4.91475300	-1.07133200	-2.18888900
C	-6.58787400	0.07127900	1.18291300
H	-4.65113000	0.71459500	1.73880600
C	-7.36577600	-0.51439800	0.18023400
O	-2.68062900	0.10926200	-1.58864000
O	-7.14375900	0.49261500	2.34120500
H	-8.09464900	0.32454100	2.32970800
O	-8.66555900	-0.66878200	0.37846100
H	-9.04540100	-1.07950100	-0.41903100
O	-7.55312300	-1.45654100	-1.92700100

C–HB$_2$ – Radical (4-O)

0 2	x	y	z
C	-1.85005200	2.00224900	0.41832500
C	-3.85161400	0.66319600	0.37484500
C	-3.16930500	-0.49050800	0.01275800
C	-1.71238500	-0.43855900	-0.19526700
C	-1.07580400	0.85163500	-0.02204900
H	-5.75409500	1.56646500	0.84209200
C	-5.23166900	0.66128700	0.56149500
C	-3.88706900	-1.67933800	-0.16074300
C	-5.25496600	-1.71004700	0.02255700
C	-5.90673100	-0.52884500	0.37991900
H	-3.34224900	-2.57217500	-0.44077500
H	-5.82354000	-2.62206500	-0.10599000
O	-3.21208500	1.84661300	0.56965800
O	-1.43552800	3.10499500	0.68072100
O	-1.10647600	-1.47178700	-0.51377500
C	0.31727600	1.01074000	-0.24596800
C	1.03517600	2.31708000	-0.14811200
H	0.47103400	3.08801300	-0.66710600
H	1.09676400	2.61606200	0.89989800
H	2.03534500	2.23605300	-0.56421500
O	-7.27240600	-0.54228300	0.62018700
C	-8.10741100	-0.64629900	-0.45393900
C	-9.53430100	-0.68811900	-0.00764900
H	-9.76045200	0.20891400	0.56997700
H	-10.18486400	-0.75200400	-0.87521900
H	-9.68349700	-1.55134400	0.64233300
O	-7.70454200	-0.68914700	-1.58176000
N	1.02328900	-0.06522200	-0.57613200
H	0.52355700	-0.96390600	-0.61466000

N	2.39220500	-0.05917100	-0.70689500
C	3.17345100	-0.02248000	0.42395700
H	2.74296800	-0.44429900	-1.57357400
C	4.64209800	-0.17557300	0.17617800
C	5.38735200	-0.74103900	1.22250500
C	5.21706900	0.24796500	-1.03184700
C	6.73942400	-0.92043700	1.05685400
H	4.89776700	-1.03825400	2.14049400
C	6.57249000	0.08454300	-1.21252000
H	4.63575300	0.73090600	-1.80685400
C	7.39725900	-0.51646000	-0.17598100
O	2.70530400	0.15259100	1.52796700
O	7.18630300	0.47318700	-2.32583200
H	8.13140600	0.26522000	-2.23778200
O	8.62140500	-0.67080000	-0.33699800
O	7.50386600	-1.46468200	2.00098000
H	8.41720600	-1.49227000	1.67147700

C–HB$_2$ – Radical (N)

0 2	x	y	z
C	2.06500900	2.41020200	-0.00506700
C	3.91521200	0.88454900	-0.19565600
C	3.10126600	-0.23919700	-0.24289600
C	1.64228300	-0.09247000	-0.16323200
C	1.12543100	1.27890500	-0.04166700
H	5.92449400	1.66425100	-0.22672400
C	5.30060500	0.78096500	-0.26338900
C	3.69409100	-1.50290300	-0.36621700
C	5.06478900	-1.63406300	-0.44037100
C	5.85031600	-0.48009100	-0.38432100
H	3.04914200	-2.37173600	-0.40468200

H	5.53478700	-2.60352300	-0.54163600
O	3.40769700	2.14247900	-0.07978800
O	1.77415100	3.57367400	0.08590000
O	0.92067900	-1.08905600	-0.19871600
C	-0.24335600	1.55374400	0.03688100
C	-0.84675600	2.91502900	0.16247400
H	-0.45359900	3.40917100	1.05077200
H	-0.55367700	3.52725400	-0.69078200
H	-1.92728100	2.84166300	0.21841900
O	7.22445100	-0.56528500	-0.51826000
C	7.93495500	-1.20162900	0.46011100
C	9.38988500	-1.22856400	0.11614000
H	9.75704300	-0.20539500	0.02509200
H	9.93612800	-1.75599500	0.89292600
H	9.52667400	-1.72035700	-0.84756700
O	7.41888100	-1.64887300	1.44398500
N	-1.10309100	0.49916300	-0.00425400
H	-0.68992600	-0.44949400	-0.08122100
N	-2.40114000	0.63505100	0.04334500
C	-3.03845000	-0.62049200	-0.01375100
C	-4.51488600	-0.54265900	0.01608900
C	-5.22845700	-1.74488900	-0.01514200
C	-5.17725100	0.68566700	0.06868600
C	-6.61088200	-1.70888400	0.00780800
H	-4.69673000	-2.68794600	-0.05723900
C	-6.56398700	0.70954900	0.08886500
H	-4.63037400	1.61817000	0.09289000
C	-7.28216000	-0.48444900	0.05907600
O	-2.41598500	-1.66720800	-0.08081600
O	-7.21955900	1.89661100	0.13759700
H	-8.17161000	1.73432400	0.14322600
O	-8.63369900	-0.40369300	0.08199300

H	-9.01330600	-1.29205800	0.05574200
O	-7.42762600	-2.79661400	-0.01552500
H	-6.92002100	-3.61403600	-0.06035100

C–HB$_2$ – Anion (3-O/5–O)

-1 1	x	y	z
C	-1.85437900	2.00944100	0.35879200
C	-3.84975500	0.65921100	0.35174300
C	-3.16681200	-0.48882900	-0.02621900
C	-1.71330900	-0.42659900	-0.26087600
C	-1.08050600	0.86321500	-0.08795100
H	-5.74824000	1.54779000	0.86208800
C	-5.22557700	0.64675600	0.56866000
C	-3.87948700	-1.68243100	-0.18601700
C	-5.24307600	-1.72377500	0.02727500
C	-5.89536600	-0.54826600	0.40121400
H	-3.33407700	-2.57093100	-0.47853300
H	-5.80781200	-2.63985400	-0.08910400
O	-3.21472300	1.84645000	0.53201400
O	-1.44627000	3.11782900	0.61044000
O	-1.10942900	-1.45639700	-0.59672800
C	0.31745600	1.02455900	-0.30966900
C	1.03226100	2.33172800	-0.19767700
H	0.47406300	3.10327700	-0.72234900
H	1.07985100	2.62722100	0.85181700
H	2.03812900	2.24899300	-0.59929600
O	-7.25555100	-0.57419500	0.67344400
C	-8.11435700	-0.65209700	-0.38308100
C	-9.53110800	-0.70204200	0.09412300
H	-9.75040200	0.19950600	0.66775000
H	-10.19991100	-0.77661400	-0.75856400
H	-9.66080500	-1.55941000	0.75554200

O	-7.73747400	-0.66768600	-1.52074600
N	1.02568500	-0.04287100	-0.64749400
H	0.52728800	-0.94080600	-0.69162900
N	2.39797900	-0.03445300	-0.74754400
C	3.15585900	-0.00863400	0.41554900
H	2.76374300	-0.47427100	-1.58029900
C	4.61682800	-0.17323500	0.20761700
C	5.35748900	-0.68991600	1.27542300
C	5.21312300	0.18158700	-1.00545700
C	6.75087300	-0.89443500	1.16174000
H	4.85552500	-0.94978200	2.19988000
C	6.59149400	0.00000900	-1.13784100
H	4.66375500	0.63842900	-1.81884800
C	7.32411000	-0.52476100	-0.09301900
O	2.62390300	0.16809700	1.49476400
O	7.20735900	0.35869800	-2.30737600
H	8.15110900	0.17792400	-2.21923200
O	8.67023800	-0.70166200	-0.23165300
H	8.93731800	-1.06820200	0.63258900
O	7.53822900	-1.37000100	2.05927800

C–HB_2 – Anion (4-O)

-1 1	x	y	z
C	-1.84687300	2.06993500	-0.09333700
C	-3.85067900	0.76128900	0.18366600
C	-3.17178500	-0.44463900	0.08080600
C	-1.71611100	-0.44260000	-0.15026800
C	-1.07936400	0.84982200	-0.27414200
H	-5.74700800	1.75091300	0.46397300
C	-5.22870000	0.80439900	0.38339200
C	-3.89181400	-1.64023600	0.18701300

C	-5.25714400	-1.62681300	0.39085900
C	-5.90592600	-0.39409000	0.48298600
H	-3.35008500	-2.57436300	0.10691400
H	-5.82500100	-2.54401400	0.47717000
O	-3.20973300	1.95596000	0.10184900
O	-1.43241900	3.20464700	-0.08683200
O	-1.11565700	-1.52484900	-0.23779300
C	0.31875800	0.95128700	-0.53821100
C	1.03262500	2.24726400	-0.74504400
H	0.46524900	2.87400500	-1.42894200
H	1.09262500	2.78125800	0.20456500
H	2.03354200	2.06994800	-1.12787800
O	-7.26488600	-0.34114000	0.75146600
C	-8.13150000	-0.80758900	-0.19290800
C	-9.54155300	-0.70761000	0.29648400
H	-9.77545500	0.33678800	0.50781300
H	-10.21784100	-1.09927100	-0.45792800
H	-9.64316000	-1.26782200	1.22663700
O	-7.76729500	-1.22048300	-1.25738800
N	1.02214900	-0.16534400	-0.62461500
H	0.52296100	-1.04702200	-0.45058300
N	2.39283000	-0.18187000	-0.76278000
C	3.17240100	0.09513400	0.36751100
H	2.71812000	-0.87168800	-1.42518700
C	4.61323800	-0.12749600	0.20375800
C	5.40012700	-0.17025500	1.36781100
C	5.22015200	-0.29236900	-1.05625800
C	6.75639600	-0.39225800	1.27054000
H	4.93590200	-0.04025400	2.33836600
C	6.57973000	-0.50796300	-1.13257700
H	4.65987700	-0.21479700	-1.98130100
C	7.40711900	-0.57174400	0.02063400

O	2.64108600	0.52796500	1.37653500
O	7.21506700	-0.65620800	-2.33361900
H	8.14993500	-0.78118300	-2.10082000
O	8.67127600	-0.77256100	-0.06442400
O	7.55673200	-0.45375400	2.37715400
H	8.44753000	-0.61940700	2.02708600

C–HB$_2$ – Anion (N)

-1 1	x	y	z
C	2.08981900	2.34841700	-0.22348200
C	3.95653900	0.82630100	-0.29166700
C	3.13058600	-0.28930000	-0.32381600
C	1.66372800	-0.11322900	-0.28463200
C	1.17949500	1.23186800	-0.15519800
H	5.97733700	1.58326300	-0.28693800
C	5.34500500	0.70504900	-0.30896800
C	3.71303000	-1.56011200	-0.38070100
C	5.08668300	-1.70953500	-0.40255700
C	5.88243000	-0.56454100	-0.36449700
H	3.05832900	-2.42228200	-0.40763800
H	5.55118400	-2.68636700	-0.44993100
O	3.45258500	2.08439100	-0.25514900
O	1.81624200	3.52937800	-0.26829400
O	0.93474500	-1.11975100	-0.34510900
C	-0.23443200	1.50329700	0.00282900
C	-0.77546500	2.85316400	0.36682500
H	-0.12115100	3.34025600	1.08523800
H	-0.81119700	3.49203000	-0.51838100
H	-1.77916100	2.74073100	0.76617900
O	7.26469500	-0.68769400	-0.44405600
C	7.93127000	-1.08529200	0.67486300

C	9.39685000	-1.20831200	0.39906500
H	9.77717700	-0.25974300	0.01856200
H	9.91709600	-1.48646400	1.31118400
H	9.55433700	-1.96534400	-0.37061800
O	7.37790600	-1.29517100	1.71774000
N	-1.09292100	0.52649600	-0.13788800
H	-0.72859300	-0.41640500	-0.33426200
N	-2.45018000	0.66690700	-0.00407100
C	-3.04929100	-0.51677100	-0.15538300
C	-4.54919500	-0.47025800	-0.03496200
C	-5.24880500	-1.67378600	-0.12448400
C	-5.23764400	0.72723500	0.16303000
C	-6.63195300	-1.67470900	-0.01281000
H	-4.70426300	-2.59755800	-0.27924100
C	-6.62103500	0.71700400	0.27195400
H	-4.70330900	1.66533500	0.23351400
C	-7.32298200	-0.48342800	0.18479600
O	-2.47902400	-1.60903500	-0.37723900
O	-7.29772800	1.88321900	0.46436200
H	-8.24177200	1.69006900	0.52491100
O	-8.68086100	-0.43448300	0.30004600
H	-9.03811300	-1.32883900	0.23131200
O	-7.41993000	-2.78877400	-0.08239000
H	-6.88303900	-3.57750500	-0.21068000

C–HB$_1$ – Radical Cation

1 2	x	y	z
C	-1.75729200	1.99655800	-0.45536800
C	-3.77269100	0.78110500	0.05370300
C	-3.13688300	-0.45219200	0.02774400

C	-1.69945400	-0.52252000	-0.27800900
C	-1.02576300	0.73794600	-0.53932900
H	-5.61865000	1.85757400	0.34588100
C	-5.13340000	0.89065400	0.32682900
C	-3.88367800	-1.60756200	0.28877600
C	-5.23234600	-1.52722900	0.56914800
C	-5.83827900	-0.26909200	0.58025600
H	-3.37538400	-2.56341800	0.26839900
H	-5.81911600	-2.41176600	0.77945500
O	-3.10532100	1.94248600	-0.18017400
O	-1.31162200	3.10840200	-0.59755300
O	-1.13468300	-1.62409200	-0.30558100
C	0.35349000	0.77304100	-0.84805800
C	1.11018200	2.02554000	-1.14848400
H	0.57735200	2.59790600	-1.90513100
H	1.14997100	2.64766300	-0.25314900
H	2.11649500	1.80514500	-1.49116000
O	-7.17395800	-0.14069000	0.92167100
C	-8.11292300	-0.69788900	0.10176700
C	-9.48324900	-0.50416000	0.66869700
H	-9.66304400	0.55857300	0.83358000
H	-10.22046000	-0.91168700	-0.01721500
H	-9.54638800	-1.00841900	1.63415200
O	-7.82970800	-1.25037400	-0.92272300
N	1.01449300	-0.38575100	-0.88610300
H	0.49176800	-1.24040300	-0.64906900
N	2.37689200	-0.47356000	-1.04284100
C	3.19371900	-0.15962900	-0.01405300
H	2.70247000	-0.88855700	-1.90425100
C	4.65135900	-0.36801400	-0.16981400
C	5.29799200	-0.92040100	-1.27992000
C	5.44048700	0.02739600	0.91509400

C	6.68644000	-1.09257700	-1.36115800
H	4.72234700	-1.24180700	-2.13943800
C	6.87519600	-0.13858000	0.84714700
C	7.49315100	-0.70996300	-0.31464100
O	2.74430800	0.32322700	1.02938700
O	7.64230600	0.21795200	1.83027700
H	7.14036900	0.59000300	2.57998300
O	8.80431500	-0.85963100	-0.36173400
H	9.23622400	-0.54578400	0.44669000
H	7.13391600	-1.52614700	-2.24463800
O	5.00034300	0.54850000	2.02072700
H	3.98825900	0.59547600	1.92217000

C–HB$_2$ – Radical Cation

1 2	x	y	z
C	-1.86102900	1.96280800	0.54856700
C	-3.87540900	0.64995000	0.41420200
C	-3.21163800	-0.47202600	-0.06298400
C	-1.75740400	-0.41703500	-0.28333900
C	-1.10269400	0.84599500	0.00120200
H	-5.75878900	1.52476500	0.99634800
C	-5.25201900	0.64447900	0.62329500
C	-3.94604500	-1.63275800	-0.33367600
C	-5.31083500	-1.66677700	-0.13126900
C	-5.94374500	-0.51727300	0.34549100
H	-3.41650900	-2.50175400	-0.70361300
H	-5.89084200	-2.55757200	-0.33503500
O	-3.22060900	1.80521700	0.70648600
O	-1.43112200	3.03516500	0.89663500
O	-1.16557900	-1.42200800	-0.70169800
C	0.28868400	1.00647800	-0.21166200
C	1.02477200	2.28778300	0.00744900

H	0.47612600	3.10746300	-0.45039300
H	1.07629100	2.49719900	1.07727000
H	2.02809900	2.23311800	-0.40477600
O	-7.30280300	-0.53598700	0.61477400
C	-8.16624100	-0.61616400	-0.43940400
C	-9.58037300	-0.66851000	0.04441200
H	-9.79688200	0.22882900	0.62557100
H	-10.25318400	-0.73749400	-0.80556300
H	-9.70663100	-1.53072200	0.70031100
O	-7.79359900	-0.63090100	-1.57808700
N	0.97859600	-0.04578600	-0.64760500
H	0.46489600	-0.92985100	-0.77134000
N	2.34802800	-0.05180400	-0.76543500
C	3.11606900	-0.10710300	0.36282100
H	2.71135300	-0.31912600	-1.67120300
C	4.59611600	-0.21501300	0.11499400
C	5.32711600	-0.94095900	1.09720400
C	5.18474000	0.40573400	-0.96912000
C	6.67625600	-1.07521000	0.96588100
H	4.79744500	-1.38224100	1.93211500
C	6.56469900	0.29650500	-1.12349100
H	4.62660400	0.99584500	-1.68357900
C	7.32452000	-0.45662500	-0.15076000
O	2.66683700	-0.03187300	1.48315600
O	7.17776300	0.86918200	-2.12593800
H	8.13754300	0.71884500	-2.11063100
O	8.60427800	-0.53376500	-0.34307600
H	9.05121000	-1.05107900	0.35364500
O	7.52750100	-1.72541600	1.76069800
H	7.09231500	-2.13567700	2.51967300
